# Fuling-Guizhi Herb Pair in Coronary Heart Disease: Integrating Network Pharmacology and In Vivo Pharmacological Evaluation

**DOI:** 10.1155/2020/1489036

**Published:** 2020-05-17

**Authors:** Bailu Duan, Lintao Han, Shuping Ming, JingJing Li, Qiong Wang, Dongning Zhang, Guangyu Tian, Fang Huang

**Affiliations:** ^1^College of Basic Medicine, Hubei University of Chinese Medicine, Wuhan 430065, China; ^2^Pharmacy School, Hubei University of Chinese Medicine, Wuhan 430065, China; ^3^First Clinical College, Hubei University of Chinese Medicine, Wuhan 430065, China; ^4^Department of Neurology, Hubei Provincial Traditional Chinese Medical Hospital, Wuhan 430065, China

## Abstract

The Fuling (*Poria cocos*)-Guizhi (*Cinnamomi ramulus*) herb pair (FGHP) is a commonly used traditional Chinese herbal formula with coronary heart disease (CHD) treatment potential. However, the mechanism of FGHP in the treatment of CHD was still unclear. In this study, the action targets and underlying mechanism of FGHP against CHD were successfully achieved by combined network pharmacology prediction with experimental verification. 76 common targets were screened out by overlapping the chemical-protein data of FGHP and CHD-related targets. Then, two key targets were further selected for verification by using western blot analysis after analyzing PPI, GO function, and KEGG pathway. Results indicated FGHP could alleviate CHD syndromes and regulate inflammatory responses in acute myocardial ischemia rats, and the reduction of expression of TNF-*α* and IL-6 in myocardial tissue would be one of its possible underlying mechanisms. Our work demonstrated that network pharmacology combined with experimental verification provides a credible method to elucidate the pharmacological mechanism of FGHP against CHD.

## 1. Introduction

Coronary heart disease (CHD) is a common type of heart disease and one of the most common causes of human deaths in the world [1, 2]. The World Health Organization (WHO) estimates that approximately 17.9 million people die of cardiovascular diseases in the world every year [3] and 7.4 million die of CHD [4]. It is well-known that CHD is an archetypical multifactorial disease [5]. Single-target drugs usually fail as a cure for this multifactorial disease [6]. In addition, repeated or continuous high-dose of a single specific drug can produce drug resistance [6, 7]. Therefore, the discovery of new multitarget drugs is highly necessary.

Traditional Chinese medicine (TCM), mainly Chinese herbal medicine, has been used for thousands of years to treat CHD and its related diseases, as it is characterized by multiple components, and it can treat diseases through multiple pathways and targets [8, 9]. Herb pairs (combination of two herbs) are the simplest form of herbal combination and compatibility [10], which have therapeutic features and clinical significance in Chinese herbal medicine. The Fuling-Guizhi herb pair (FGHP) is a famous formula originated from Linggui Zhugan Decoction, a well-known Chinese medicinal formula consisting of four Chinese herbal medicines—*Poria cocos* (Chinese name, Fuling), *Cinnamomi ramulus* (Chinese name, Guizhi), *Atractylodes macrocephala* Koidz. (Chinese name, Baizhu), and *Licorice* (Chinese name, Gancao), recorded in the classic ancient book “JinGuiYaoNue” written by Zhongjing Zhang. FGHP has been commonly used clinically for the treatment of CHD. However, the mechanism by which FGHP helps to treat CHD remains unclear, which impedes its further clinical application and spread in some degree.

Network pharmacology recently has become a new and a powerful approach to systematically reveal the principles and function of a complex biological system along with the rapid progress of bioinformatics [11, 12], and it has become an efficient way to preliminarily observe action at the system level and to explore the potential pharmacological mechanism of TCM at the molecular level [12, 13].

Therefore, network pharmacology-based study was adopted in this study to investigate underlying action mechanism of FGHP in CHD. First, the potential action of FGHP was predicted using a network pharmacological method. Subsequently, an isoproterenol- (ISO-) induced acute myocardial ischemia (AMI) experiment was carried out on rats to confirm the network analysis-based data.

## 2. Materials and Methods

### 2.1. Network Pharmacology-Based Analysis

#### 2.1.1. Active Component Screening

Firstly, chemical compounds in Fuling (FL) and Guizhi (GZ) were obtained from the Encyclopedia of Traditional Chinese Medicine (ETCM, http://www.nrc.ac.cn: 9090/ETCM), the Traditional Chinese Medicine Integrated Database (TCMID, http://www.megabionet.org/tcmid/), and the Traditional Chinese Medicine Systems Pharmacology Database (TCMSP, http://tcmspw.com/tcmsp.php). The active components were the filtered by combining oral bioavailability (OB) ≥30% and drug-likeness (DL) index ≥0.18 as suggested by the TCMSP database.

#### 2.1.2. Target Prediction of FGHP against CHD

SWISS (http://www.swisstargetprediction.ch/) and STITCH (http://stitch.embl.de/) databases were used to identify the potential human protein targets of FGHP's active compounds. At the same time, CHD-related human genes were downloaded from OMIM (https://omim.org/) and DisGeNET (https://www.disgenet.org/) databases. Then, the candidate targets of FGHP against CHD were obtained by overlapping the above targets with a Venn diagram (http://bioinfogp.cnb.csic.es/tools/venny/index.html).

#### 2.1.3. Gene Ontology and Pathway Enrichment Analysis

The gene ontology (GO) and Kyoto Encyclopedia of Genes and Genomes (KEGG) enrichment analysis of the candidate targets were carried out using the DAVID (https://david.ncifcrf.gov/) to obtain the related functions and pathways.

#### 2.1.4. Protein-Protein Interaction (PPI) Data

The protein-protein interaction (PPI) data were gathered from STRING (https://string-db.org/) database, which supplies information regarding the predicted and verified experimental interactions of proteins [14], with species simply limited to “*Homo sapiens*” and interaction score >0.7 (high confidence). The top 10 proteins with higher levels of correlation were collected as the center targets for FGHP against CHD.

#### 2.1.5. Network Construction

Based on these results, a compound-target-pathway network composed of compounds, corresponding targets, and their related potential pathways was constructed and displayed using Cytoscape 3.7.1 (http://cytoscape.org/), which is a software package for visualizing network analysis [15].

### 2.2. Evaluation of Anti-CHD Properties of FGHP In Vivo

#### 2.2.1. Animals and Drugs

Male SD rats weighing 220–250 g (6–8 weeks) were obtained from the Hubei Experiment Animal Research Center (Wuhan, China). All rats were housed in the SPF laboratory animal room, Hubei University of Chinese Medicine (Wuhan, China), and maintained in suitable conditions with a 12 h light/dark cycle and room temperature at 22 ± 2°C and 50 ± 10% humidity. They had free access to food and water. The study protocol was approved by Animal Ethics Committee of Hubei University of Chinese Medicine (HUCMS-201903001). Fuling and Guizhi were purchased from TCM pharmacy of Wuhan Hospital of Traditional Chinese Medicine (Wuhan, China). The FGHP was obtained by mixing the two herbs (Fuling and Guizhi) at a mass ratio of 1 : 1 and then grinding them into powder. Propranolol tablets were purchased from Ya Bang Pharmaceutical Factory (Changzhou, China). Isoproterenol (ISO) hydrochloride was purchased from Southwest Pharmaceutical Co., Ltd. (Chongqing, China).

#### 2.2.2. Experimental Process

Rats were randomly divided into the control group and three administration groups (*n* = 6 per group): ISO, ISO + FGHP (1.8 g/kg), and ISO + Propranolol (Pro, 10 mg/kg). The rats in the two ISO+ groups were pretreated by oral gavage administration with propranolol or FGHP, while the rats in the control group and the ISO group were pretreated by oral gavage administration with equal volumes of 0.9% saline once a day for 2 weeks. Afterwards, the rats in all three administration groups were subcutaneously injected with ISO (85 mg/kg) for two consecutive days [16, 17]. FGHP dosage for the rats was calculated in proportion to human doses (20 g/day per adult) using body surface area conversion.

At the end of the experiment, electrocardiograms (ECGs) were recorded under pentobarbital anesthesia using a BL-420F biological data acquisition and analysis system. Blood was collected via the abdominal aorta. The serum was separated by centrifugation at 4°C and 3,000 rpm for 15 min and subsequently stored at −20°C for biochemical analysis. The heart tissues were dissected and immediately frozen in liquid nitrogen or fixed in 10% paraformaldehyde for western blot or histological analysis.

#### 2.2.3. Biochemical Analysis

The serum creatine kinase-MB (CK-MB) and lactate dehydrogenase (LDH) levels were measured by using commercial kits and following the product instructions (CUSABIO BIOTECH Co., Ltd., Wuhan, China).

#### 2.2.4. Histopathological Analysis

The myocardial tissues were dissected and then fixed in 10% paraformaldehyde overnight followed by processing of dehydrating, paraffin embedding, sectioning, and staining (H&E). Morphological structure of each specimen was examined and photographed by using a light microscope (BH-2, Olympus, Japan).

#### 2.2.5. Western Blot Analysis

The total protein from the myocardial tissues were extracted as described previously[17]. Next, the protein was separated on 12% SDS-PAGE and electrophoretically transferred onto PVDF membrane. After blocking with 5% nonfat milk in TBS-0.1% Tween-20 (TBST) for 1 hour, the membranes were incubated with the following primary antibodies: anti-IL-6 (#12912), anti-TNF-*α* (#6954) (CST, Massachusetts, USA), and anti-*β*-actin (BOSTER, Wuhan, China) at 1 : 1000 dilution at 4°C overnight. Following washing in TBST, the membranes were incubated with corresponding horseradish peroxidase-conjugated secondary antibodies. Then, the protein bands were visualized with a BeyoECL Plus (P0018M, Beyotime, Shanghai, China). Densitometry analysis of each band was performed using the BioRad Quantity One software. *β*-Actin protein was used as the internal control for semiquantitative analysis.

### 2.3. Statistical Analysis

Data were expressed as the mean ± SEM. GraphPad Prism 5 software (San Diego, CA, United States) was used for the statistical analysis and graphics. Unpaired *t*-test was used to analyze statistical comparisons between two groups. Multiple comparisons were assessed by one-way ANOVA. *P* < 0.05 was assumed as statistically significant.

## 3. Results

### 3.1. Results of Network Pharmacology-Based Analysis

#### 3.1.1. Active Components and Target Identification of FGHP

Totally, 33 components of Fuling (FL) and 220 components of Guizhi (GZ) were obtained from the ETCM, TCMID, and TCMSP (Table S1). All active components should satisfy the filtering rules, OB index ≥30% and DL value ≥0.18. After screening, a total of 18 compounds of FGHP were collected, including 11 in FL and 7 in GZ (as shown in Table 1). By fishing for targets, 277 potential targets were found for FGHP. Detailed information of the targets is provided in Table S2.

#### 3.1.2. Targets of FGHP against CHD

**Figure 1:**
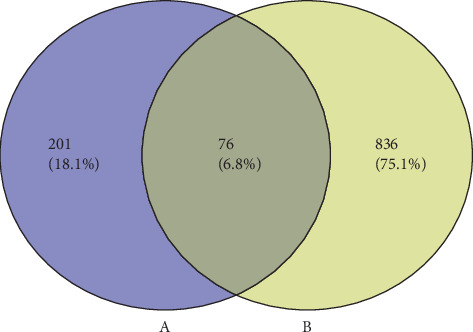
Overlapping targets between FGHP (A) and CHD (B).

A total of 912 CHD-related targets were obtained from the DisGeNET and OMIM. The detailed information of the targets is shown in Table S3. Then, the predictive targets of FGHP were overlapped with the CHD-related targets. Totally, 76 candidate targets of FGHP against CHD were identified, excluding any duplicate targets (Figure 1). These targets are listed in Table S4.

#### 3.1.3. GO and Pathway Enrichment Analysis

**Figure 2:**
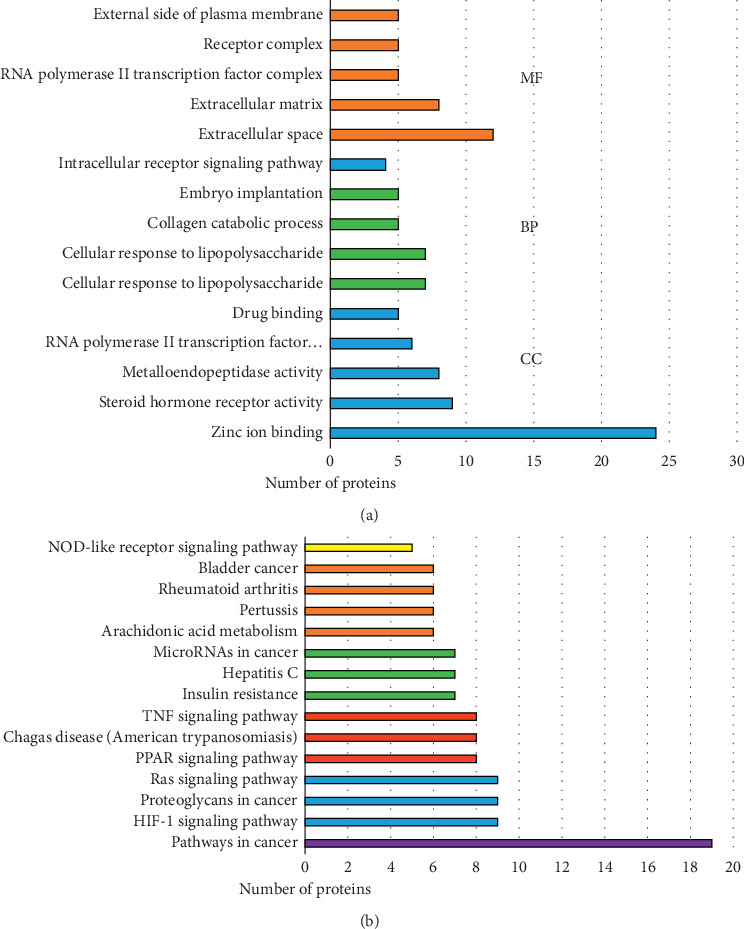
GO (a) and pathway (b) enrichment analysis by DAVID.

To explore the multiple mechanisms of FGHP on CHD from a systematic level, gene ontology (GO) analysis and KEGG pathway enrichment of the 76 candidate targets were performed. In total, 96 enriched GO terms and 48 pathways were identified (*P* < 0.05, Table S5). Top 5 GO functional categories in biological process, cellular component, and molecular function and 15 remarkable pathways were selected and are presented in Figures 2(a) and 2(b).

#### 3.1.4. PPI Network of Target Genes

**Figure 3:**
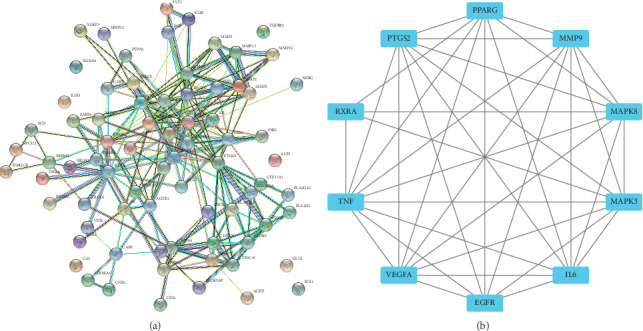
PPI network of targets for FGHP against CHD (a). Top 10 targets in PPI network (b).

The STRING tool was employed to obtain PPI relationships for the 76 target genes. With a confidence score of >0.7 was selected, the network of PPI relationships has 76 nodes and 237 edges (Figure 3(a)). The top 10 proteins with higher levels of connectivity were collected as the center targets for FGHP against CHD (Figure 3(b)).

The center target genes, which might play an important role in CHD progression, were peroxisome proliferator activated receptor gamma (PPARG), matrix metalloproteinase 9 (MMP9), mitogen-activated protein kinase 8(MAPK8), mitogen-activated protein kinase 3(MAPK3), interleukin 6 (IL6), epidermal growth factor receptor (EGFR), vascular endothelial growth factor A (VEGFA), tumor necrosis factor (TNF), retinoid X receptor alpha (RXRA), and prostaglandin-endoperoxide synthase 2 (PTGS2).

#### 3.1.5. Compound-Target-Pathway Network

**Figure 4:**
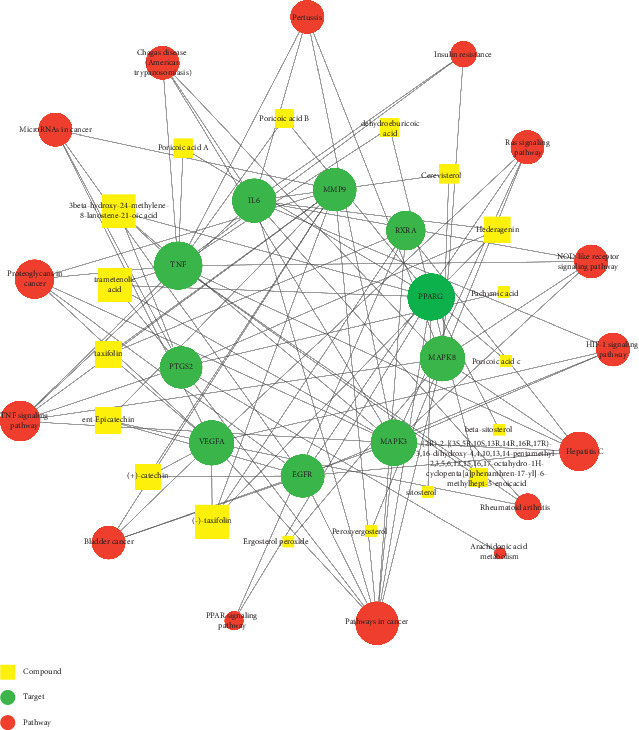
The component-target-pathway network. There is a positive proportional relationship between the node size and the edge count.

Based on the all above information, we constructed a compound-target-pathway network (Figure 4) to explain the mechanism of FGHP in treating CHD. This network had 43 nodes (18 compounds, 10 targets, and 15 pathways) and 102 edges, in which yellow squares, green circles, and red circles correspond to active components, their corresponding target proteins, and potential pathways involved, respectively.

### 3.2. Results of Pharmacodynamic Study of FGHP

#### 3.2.1. The Effect of FGHP on ECG

**Figure 5:**
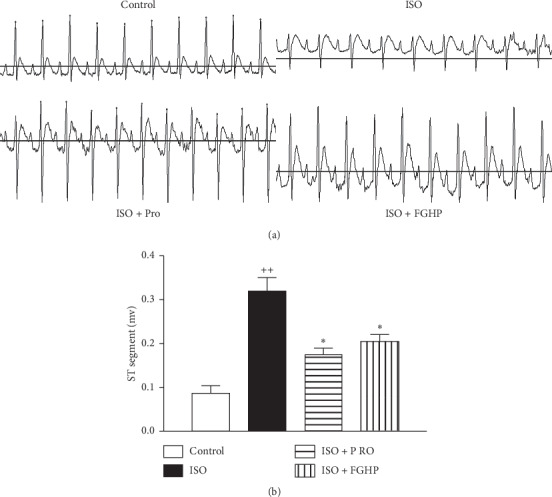
Effects of FGHP on ECG in AMI rats. ECG recordings (a) and bar graph of ST segment (b). ^++^*P* < 0.01 vs. the control group; ^*∗*^*P* < 0.05 vs. the ISO group. Values are presented as mean ± SEM.

As shown in Figure 5, the control group showed normal ECG readings (Figure 5(a)), whereas rats injected with 85 mg/kg ISO showed a marked increase in the ST segment, when compared with the normal controls. These results suggest that the acute myocardial ischemia (AMI) model was successfully established. Though FGHP and propranolol pretreatments could not completely inhibit the ISO-induced elevation of the ST segment, obvious decreases in the ST segment were observed in FGHP and Pro pretreated groups compared with the ISO group (Figure 5(b)).

#### 3.2.2. Effects of FGHP on Serum Cardiac Markers

**Figure 6:**
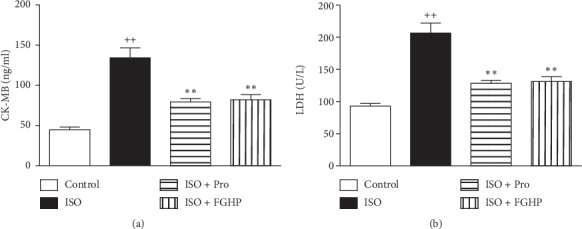
Effects of FGHP on CK-MB (a) and LDH (b) in AMI rats. ^++^*P* < 0.01 vs. the control group; ^*∗∗*^*P* < 0.01 vs. the ISO group. Values are presented as mean ± SEM.

The serum concentrations of creatine kinase‐MB (CK-MB) and lactic dehydrogenase (LDH) in the ISO group were significantly increased than those in the control group (*P* < 0.01). Pretreatment with FGHP and propranolol markedly inhibited the elevation in the levels of serum CK-MB and LDH of the ISO group (*P* < 0.01) as shown in Figures 6(a) and 6(b).

#### 3.2.3. The Effect of FGHP on Histopathological Features

**Figure 7:**
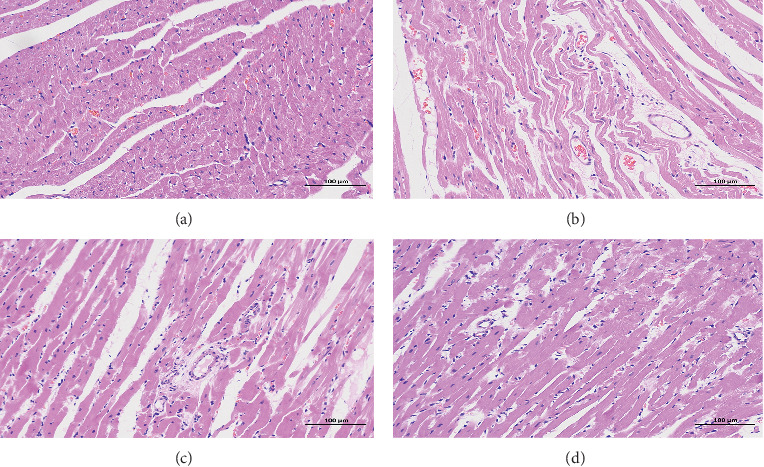
Effects of FGHP on histopathological features in AMI rats. Representative H&E staining images of heart paraffin sections in the various groups. Original magnification ×200. (a) Control. (b) ISO. (c) ISO + Pro. (d) ISO + FGHP.

Compared to the control group (Figure 7(a)), the ISO group rats (Figure 7(b)) showed more frequently pathological changes, such as myocardial cell swelling, separation of cardiac muscle fibers, interstitial edema, and inflammatory cell infiltration. Such histological changes were significantly alleviated in the propranolol (Figure 7(c)) and FGHP (Figure 7(d)) pretreatment groups compared to the ISO group.

### 3.3. Experimental Validation of the Network Pharmacology

**Figure 8:**
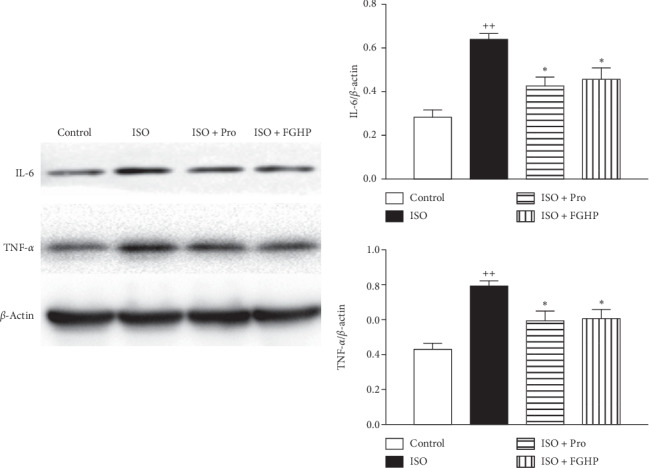
Effects of FGHP on TNF-*α* and IL-6 in cardiac muscle in AMI rats by western blot analysis. ^++^*P* < 0.01 vs. the control group; ^*∗*^*P* < 0.05 vs. the ISO group. Values are presented as mean ± SEM.

Based on the network pharmacology analysis, to verify the results derived using the PPI and compound-target-pathway network, we chose the high two related targets (TNF-*α* and IL-6) for experimental validation. As shown in Figure 8, the protein levels of TNF-*α* and IL-6 were significantly increased in the ISO group compared with those in the control group (*P* < 0.01). Pretreatment with FGHP or propranolol showed that TNF-*α* and IL-6 expression decreased obviously in the ISO + FGHP or the ISO + Pro groups compared with the ISO group (*P* < 0.05).

## 4. Discussion

CHD is a complex disease and the final manifestation of various pathological insults [5, 18]. Developing novel comprehension multicomponent synergy strategies are vital for conquering complex and multifactorial diseases [19]. It is believed multicomponent and multitarget drugs can effectively reduce side effects and improve adaptive resistance, thus improving the possibility of overcoming diseases [7, 19]. It is generally known that for over 5,000 years, traditional Chinese medicine (TCM) derived from numerous herbal formulas or herb pairs to form multiingredient herbal medicine was used to cure diseases [20, 21]. However, it is actually recognized that the intricacy of ingredients, multiple targets, pathways, and mechanisms still restrains the development of TCMs.

The advent of network pharmacology provides a novel opportunity to explore possible pharmacological mechanisms of TCMs [22]. At present, a lot of studies have made an effort to adopt network pharmacology to investigate the intricacy of ingredients, targets, pathways, and mechanisms of action of herb pairs or herbal formulas [23–25]. Thus, we used the network pharmacology method to explore the possible pharmacological mechanisms of FGHP related to CHD in the present study.

Network pharmacological analysis of FGHP showed that 18 compounds and 76 target genes were associated to CHD. According to the results of pathway enrichment, we believe that effects of FGHP against CHD may be due to the fact that FGHP can simultaneously target multiple pathways like pathways in cancer, HIF-1 signaling pathway, PPAR signaling pathway, TNF signaling pathway, and so on (shown in Figure 2(b)). Based on the results of PPI analysis, PPARG, MMP9, MAPK8, MAPK3, IL6, EGFR, VEGFA, TNF, RXRA, and PTGS2 were elected as key targets (shown in Figure 3(b)). It was proposed that the occurrence and development of CHD is widely considered as a chronic inflammatory process characterized by highly specific cytokine response [26, 27]. TNF-*α* is a key inflammatory mediator and is considered to be a major indicator of the instability of plaques during the formation of thrombi [28]. IL-6, a circulating cytokine known for its regulation of inflammatory reaction, plays a vital role in atherogenesis and thrombosis [29]. In fact, many studies have shown that various cytokines such as tumor TNF-*α* and IL-6 are involved in the pathogenesis of CHD [30–32].

FGHP originated from Linggui Zhugan Decoction, which has actually been medically used for lots of years in the treatment of several diseases, including cardiovascular diseases and metabolic diseases [33]. It was reported that Linggui Zhugan Decoction suppressed production of inflammatory mediators (IL6, TNF-*α*, and IL-1*β*) in lipopolysaccharide- (LPS-) induced cardiomyocyte injury model rats [34]. Linggui Zhugan Decoction significantly inhibited the elevation in the levels of serum IL6, IL18, and IL-10 of the chronic heart failure rats [35]. More importantly, some active ingredients in FGHP such as hederagenin and beta-sitosterol were reported to act on TNF-*α* and IL-6 to exert their anti-inflammatory properties [36, 37]. So, TNF-*α* and IL-6 were selected as the evaluation indicators to observe the therapeutic effect and possible mechanism of FGHP in ISO-induced AMI rats.

In *in vivo* studies, we attempted to study the therapeutic effect of FGHP and confirm the predicted results from network pharmacology. To this end, we tested this agent in rats with ISO-induced AMI. Our results showed that characteristic indices including CK-MB and LDH content were decreased significantly in FGHP pretreated rats compared with AMI control rats (shown in Figure 6). In addition, FGHP could ameliorate the ECG (shown in Figure 5) and myocardial tissue abnormalities in ISO-induced AMI rats to some extent (shown in Figure 7). These results indicated that FGHP could impart a therapeutic effect on acute myocardial ischemia.

Moreover, we further verify the pharmacological mechanism of FGHP on treatment for CHD by measuring the expression levels of the core and specific targets. Upregulated TNF-*α* and IL-6 expressions were detected in myocardial tissues in the ISO group rats; however, both of them were significantly reduced with FGHP pretreatment (shown in Figure 8). These findings were accompanied by decreased inflammatory cell invasion and improved pathological changes (Figure 7), indicating that suppression of TNF-*α* and IL-6 activity to modulate inflammation is at least in part responsible for FGHP exerting its cardiac protective efficacy. Further molecular mechanism studies and confirmation of the prediction of FGHP are required.

## 5. Conclusion

In summary, our study predicted and verified that FGHP could reduce protein expression of TNF-*α* and IL-6 for the suppression of inflammatory response in cardiac muscle to exert curative effect for CHD. The results partially explained the pharmacological mechanism of FGHP against CHD.

## Figures and Tables

**Table 1:** A list of the active compounds in FGHP.

No.	Compound	OB (%)	DL	Herb
1	(2R)-2-[(3S, 5R, 10S, 13R, 14R, 16R, 17R)-3,16-Dihydroxy-4,4,10,13,14-pentamethyl-2,3,5,6,12,15,16,17-octahydro-1H-cyclopenta[a]phenanthren-17-yl]-6-methylhept-5-enoic acid	30.93	0.81	FL
2	Trametenolic acid	38.71	0.8	FL
3	Cerevisterol	37.96	0.77	FL
4	Ergosterol peroxide	40.36	0.81	FL
5	3*β*-Hydroxy-24-methylene-8-lanostene-21-oic acid	38.7	0.81	FL
6	Pachymic acid	33.63	0.81	FL
7	Poricoic acid A	30.61	0.76	FL
8	Poricoic acid B	30.52	0.75	FL
9	Poricoic acid C	38.15	0.75	FL
10	Hederagenin	36.91	0.75	FL
11	Dehydroeburicoic acid	44.17	0.83	FL
12	(−)-Taxifolin	60.51	0.27	GZ
13	*β*-Sitosterol	36.91	0.75	GZ
14	Sitosterol	36.91	0.75	GZ
15	(+)-Catechin	54.83	0.24	GZ
16	ent-Epicatechin	48.96	0.24	GZ
17	Taxifolin	57.84	0.27	GZ
18	Peroxyergosterol	44.39	0.82	GZ

## Data Availability

The data used to support the findings of this study are available from the corresponding author upon request.

## Supplementary Materials

Supplementary Materials Supplementary Table S1: the detailed information of ingredients in FL and GZ. Supplementary Table S2: the detailed target information of compounds in FGHP. Supplementary Table S3: targets related to CHD. Supplementary Table S4: overlapping targets between FGHP and CHD. Supplementary Table S5: GO and pathway enrichment analysis by DAVID.
