# Supplementary material for: Fuling-Guizhi Herb Pair in Coronary Heart Disease: Integrating Network Pharmacology and In Vivo Pharmacological Evaluation
Source: Evid Based Complement Alternat Med. 2020 May 17;2020:1489036. doi: 10.1155/2020/1489036 (PMC7251461; doi:10.1155/2020/1489036)
Supplement: Supplementary Materials — Supplementary Table S1: the detailed information of ingredients in FL and GZ. Supplementary Table S2: the detailed target information of compounds in FGHP. Supplementary Table S3: targets related to CHD. Supplementary Table S4: overlapping targets between FGHP and CHD. Supplementary Table S5: GO and pathway enrichment analysis by DAVID. [file 1489036.f1.zip › 1489036.f1/Supplementary Table S1 The detailed information of ingredients in FL and GZ.docx]

**Supplementary Table S1. The detailed information of ingredients in FL and GZ**

| Compound | OB (%) | DL | Herb |
| --- | --- | --- | --- |
| (2R)-2-[(3S,5R,10S,13R,14R,16R,17R)-3,16-dihydroxy-4,4,10,13,14-pentamethyl-2,3,5,6,12,15,16,17-octahydro-1H-cyclopenta[a]phenanthren-17-yl]-6-methylhept-5-enoic acid | 30.93 | 0.81 | FL |
| 3β-hydroxylanosta-7,9(11),24-trien-21-oic acid | 24.92 | 0.8 | FL |
| trametenolic acid | 38.71 | 0.8 | FL |
| 7,9(11)-dehydropachymic acid | 35.11 | 0.81 | FL |
| tumulosic acid | 15.95 | 0.81 | FL |
| Beta-Glucan | 0.73 | 0.7 | FL |
| Cerevisterol | 37.96 | 0.77 | FL |
| (2R)-2-[(3S,5R,10S,13R,14R,16R,17R)-3,16-dihydroxy-4,4,10,13,14-pentamethyl-2,3,5,6,12,15,16,17-octahydro-1H-cyclopenta[a]phenanthren-17-yl]-5-isopropyl-hex-5-enoic acid | 31.07 | 0.82 | FL |
| Dimethyl L-malate | 8.59 | 0.03 | FL |
| ergosta-7,22E-dien-3beta-ol | 43.51 | 0.72 | FL |
| Ergosterol peroxide | 40.36 | 0.81 | FL |
| L-uridine | 23.4 | 0.11 | FL |
| (2R)-2-[(5R,10S,13R,14R,16R,17R)-16-hydroxy-3-keto-4,4,10,13,14-pentamethyl-1,2,5,6,12,15,16,17-octahydrocyclopenta[a]phenanthren-17-yl]-5-isopropyl-hex-5-enoic acid | 38.26 | 0.82 | FL |
| β-amyrin acetate | 9.11 | 0.74 | FL |
| 3beta-Hydroxy-24-methylene-8-lanostene-21-oic acid | 38.7 | 0.81 | FL |
| pachyman | 0.45 | 0.68 | FL |
| pachymic acid | 33.63 | 0.81 | FL |
| Poricoic acid A | 30.61 | 0.76 | FL |
| Poricoic acid B | 30.52 | 0.75 | FL |
| poricoic acid C | 38.15 | 0.75 | FL |
| poricoic acid D | 22.38 | 0.78 | FL |
| poricoic acid DM | 29.32 | 0.78 | FL |
| alexandrin | 20.63 | 0.63 | FL |
| hederagenin | 36.91 | 0.75 | FL |
| Tumulosic acid | 29.88 | 0.81 | FL |
| ergosterol | 14.29 | 0.72 | FL |
| Trimethyl citrate | 67.61 | 0.07 | FL |
| dehydroeburicoic acid | 44.17 | 0.83 | FL |
| 2-lauroleic acid | 31.42 | 0.04 | FL |
| caprylic acid | 16.4 | 0.02 | FL |
| Ethyl glucoside | 15.21 | 0.06 | FL |
| lauric acid | 23.59 | 0.04 | FL |
| palmitic acid | 19.3 | 0.1 | FL |
| PHB | 30.15 | 0.03 | GZ |
| protocatechuic acid | 25.37 | 0.04 | GZ |
| 1, 10, 14-trimethyl-2-pentadecanone | 21.13 | 0.1 | GZ |
| 2-Methoxycinnamic Acid | 31.79 | 0.05 | GZ |
| ST069309 | 24.02 | 0.38 | GZ |
| 2-Phenylpropenal | 30.68 | 0.02 | GZ |
| beta-Methoxystyrene | 26.54 | 0.02 | GZ |
| m-Formylphenol | 30.97 | 0.02 | GZ |
| 4-Methyl-2-(1,5-dimethyl-4-hexenyl)-3-cyclohexen-1-ol | 39.63 | 0.07 | GZ |
| DMEP | 55.66 | 0.15 | GZ |
| 2-Methyl-N-phenylmaleimide | 87.36 | 0.06 | GZ |
| β-Bisabolene | 33.46 | 0.05 | GZ |
| 694-87-1 | 48.07 | 0.03 | GZ |
| 1,2-Dibenzoylethane | 57.97 | 0.12 | GZ |
| WLN: RVO2R | 31.04 | 0.1 | GZ |
| WLN: 2OVR | 27.58 | 0.03 | GZ |
| Substance H 36 | 41.58 | 0.05 | GZ |
| 2-Coumarinate | 60.17 | 0.04 | GZ |
| WLN: VHO2R | 30 | 0.03 | GZ |
| 2-Methoxyphenylacetone | 39.61 | 0.04 | GZ |
| Hydro Cinnamicacid | 36.71 | 0.03 | GZ |
| Methyl (Z)-cinnamate | 37.2 | 0.04 | GZ |
| NK | 32.1 | 0.02 | GZ |
| (Z)-Ethyl cinnamate | 37.28 | 0.04 | GZ |
| 19894-97-4 | 49.98 | 0.06 | GZ |
| muurolene | 19.5 | 0.08 | GZ |
| Cymol | 27.2 | 0.02 | GZ |
| (L)-alpha-Terpineol | 48.8 | 0.03 | GZ |
| (1R,5R,7S)-4,7-dimethyl-7-(4-methylpent-3-enyl)bicyclo[3.1.1]hept-3-ene | 16.23 | 0.09 | GZ |
| ()-Bornyl acetate | 65.55 | 0.08 | GZ |
| (-)-alpha-Pinene | 46.25 | 0.05 | GZ |
| (-)-nopinene | 44.84 | 0.05 | GZ |
| CAM | 67.17 | 0.05 | GZ |
| PEL | 44.03 | 0.02 | GZ |
| cis-Zimtsaeure | 38.19 | 0.03 | GZ |
| Oktadekan | 9.81 | 0.09 | GZ |
| protocatechualdehyde | 38.35 | 0.03 | GZ |
| Isocaryophyllene | 27.3 | 0.09 | GZ |
| Hypnon | 48.19 | 0.02 | GZ |
| α-cubebol | 64.81 | 0.09 | GZ |
| ZINC01609418 | 21.62 | 0.07 | GZ |
| (-)-taxifolin | 60.51 | 0.27 | GZ |
| ELD | 31.2 | 0.14 | GZ |
| Tetracosane | 8.28 | 0.24 | GZ |
| Cadalin | 12.96 | 0.08 | GZ |
| D-Camphene | 34.98 | 0.04 | GZ |
| Myrcene | 24.96 | 0.02 | GZ |
| (1S,4R)-1,7,7-trimethylbicyclo[2.2.1]hept-2-ene | 39.62 | 0.04 | GZ |
| alpha-Cubebene | 16.73 | 0.11 | GZ |
| 1H-Cycloprop(e)azulen-7-ol, decahydro-1,1,7-trimethyl-4-methylene-, (1aR-(1aalpha,4aalpha,7beta,7abeta,7balpha))- | 82.33 | 0.12 | GZ |
| WLN: 2VR | 60.17 | 0.02 | GZ |
| BOX | 31.55 | 0.02 | GZ |
| Cerulignol | 62.43 | 0.04 | GZ |
| (Z)-1,3-di(phenyl)prop-2-en-1-one | 47.27 | 0.08 | GZ |
| Styrone | 38.35 | 0.02 | GZ |
| HEPTACOSANE | 8.18 | 0.36 | GZ |
| cinnamic acid | 19.68 | 0.03 | GZ |
| L-Limonen | 38.09 | 0.02 | GZ |
| Terragon | 36.59 | 0.03 | GZ |
| ()-Borneol | 81.8 | 0.05 | GZ |
| (Z,Z)-farnesol | 41.14 | 0.06 | GZ |
| Sulcatone | 26.36 | 0.01 | GZ |
| cis-Cinnamaldehyde | 27.21 | 0.02 | GZ |
| copaene | 24.08 | 0.12 | GZ |
| farnesol | 28.44 | 0.06 | GZ |
| eugenol | 56.24 | 0.04 | GZ |
| WLN: VH2R | 19.01 | 0.02 | GZ |
| alpha-Curcumene | 4.68 | 0.06 | GZ |
| anisaldehyde | 21.54 | 0.02 | GZ |
| o-Anisaldehyde | 57.94 | 0.03 | GZ |
| BZM | 18.64 | 0.09 | GZ |
| [(1S)-endo]-(-)-Borneol | 83.54 | 0.05 | GZ |
| HCI | 35.83 | 0.03 | GZ |
| Pyruvophenone | 35.93 | 0.03 | GZ |
| Cinnamyl acetate | 21.15 | 0.04 | GZ |
| O-METHOXYCINNAMALDEHYDE | 26.52 | 0.04 | GZ |
| T-Muurolol | 30.41 | 0.09 | GZ |
| ()-alpha-Longipinene | 57.47 | 0.12 | GZ |
| Sitogluside | 20.63 | 0.62 | GZ |
| beta-sitosterol | 36.91 | 0.75 | GZ |
| sitosterol | 36.91 | 0.75 | GZ |
| coumarin | 29.17 | 0.04 | GZ |
| anethole | 32.49 | 0.03 | GZ |
| (+)-catechin | 54.83 | 0.24 | GZ |
| Octadecanal | 15.38 | 0.12 | GZ |
| (-)-Caryophyllene oxide | 32.67 | 0.13 | GZ |
| Green Oil | 17.74 | 0.1 | GZ |
| ()-Terpinen-4-ol | 81.41 | 0.03 | GZ |
| (-)-alpha-cedrene | 55.56 | 0.1 | GZ |
| hexanal | 55.71 | 0.01 | GZ |
| ()-Menthol | 59.33 | 0.03 | GZ |
| DBP | 64.54 | 0.13 | GZ |
| (1R,4R)-4-isopropyl-1,6-dimethyltetralin | 17.47 | 0.08 | GZ |
| palmitic acid | 19.3 | 0.1 | GZ |
| styrene | 29.55 | 0.01 | GZ |
| WLN: VHR | 32.63 | 0.01 | GZ |
| Hyacinthin | 38.65 | 0.02 | GZ |
| ent-Epicatechin | 48.96 | 0.24 | GZ |
| p-coumaric acid | 43.29 | 0.04 | GZ |
| stearic acid | 17.83 | 0.14 | GZ |
| (1R,3R,4S)-3,4-dimethylcyclohexan-1-ol | 43.57 | 0.02 | GZ |
| 2-ethoxypropanol | 47.47 | 0.01 | GZ |
| 2,6,10,15-tetramethylheptadecane | 13.73 | 0.13 | GZ |
| beta-Bisabolene | 29.59 | 0.06 | GZ |
| (4S)-4-[(1Z)-1,5-dimethylhexa-1,4-dienyl]-1-methylcyclohexene | 28.62 | 0.06 | GZ |
| cinnamaldehyde | 31.99 | 0.02 | GZ |
| (+/-)-Isoborneol | 86.98 | 0.05 | GZ |
| Hemo-sol | 39.84 | 0.02 | GZ |
| α-Longipinene | 53.26 | 0.12 | GZ |
| beta-Selinene | 24.39 | 0.08 | GZ |
| γ-elemene | 23.79 | 0.06 | GZ |
| DIBP | 49.63 | 0.13 | GZ |
| 1,8-cineole | 39.73 | 0.05 | GZ |
| geraniol | 23.93 | 0.02 | GZ |
| NERYLACETATE | 25.94 | 0.04 | GZ |
| EIC | 41.9 | 0.14 | GZ |
| Guaiol | 38.77 | 0.09 | GZ |
| L-Bornyl acetate | 65.52 | 0.08 | GZ |
| (R)-linalool | 39.8 | 0.02 | GZ |
| Safrol | 45.34 | 0.05 | GZ |
| Moslene | 33.02 | 0.02 | GZ |
| Methyleugenol | 73.36 | 0.04 | GZ |
| ()-Aromadendrene | 55.74 | 0.1 | GZ |
| Methylcinnamate | 18.42 | 0.04 | GZ |
| (-)-beta-Phellandrene | 40.44 | 0.02 | GZ |
| o-Thymol | 43.28 | 0.03 | GZ |
| beta-Cubebene | 32.81 | 0.11 | GZ |
| CHEBI:7 | 45.2 | 0.04 | GZ |
| caprylic acid | 16.4 | 0.02 | GZ |
| (-)-Epoxycaryophyllene | 35.94 | 0.13 | GZ |
| Farnesene | 17.42 | 0.05 | GZ |
| Neryl acetate | 57.47 | 0.04 | GZ |
| delta-amorphene | 17.95 | 0.08 | GZ |
| oleic acid | 33.13 | 0.14 | GZ |
| (R)-(-)-alpha-Phellandrene | 27.51 | 0.02 | GZ |
| m-Cymol | 48.85 | 0.02 | GZ |
| MYS | 13.98 | 0.05 | GZ |
| hexadecane | 12.32 | 0.06 | GZ |
| Cedrol | 16.23 | 0.12 | GZ |
| ()-beta-Pinene | 44.77 | 0.05 | GZ |
| beta-elemene | 25.63 | 0.06 | GZ |
| Terpilene | 33.95 | 0.02 | GZ |
| (5S)-1-isopropyl-4-methylbicyclo[3.1.0]hex-3-ene | 47.13 | 0.04 | GZ |
| (R)-p-Menth-1-en-4-ol | 32.16 | 0.03 | GZ |
| .gamma.-Bisabolene | 20.78 | 0.06 | GZ |
| Hepanal | 53.83 | 0.1 | GZ |
| 58870_FLUKA | 49.01 | 0.1 | GZ |
| (1R,4S,4aR,8aR)-4-isopropyl-1,6-dimethyl-3,4,4a,7,8,8a-hexahydro-2H-naphthalen-1-ol | 31.67 | 0.09 | GZ |
| epi-10- .gamma.-Eudesmol | 28.1 | 0.1 | GZ |
| cuminal | 38.29 | 0.03 | GZ |
| (&#8722;)-Alloaromadendrene | 54.04 | 0.1 | GZ |
| o-Acetyltoluene | 38.96 | 0.02 | GZ |
| phytol | 33.82 | 0.13 | GZ |
| copaene | 29.47 | 0.12 | GZ |
| 1,4-cadinadiene | 16.73 | 0.08 | GZ |
| NON | 26.74 | 0.03 | GZ |
| (1S,4R)-fenchone | 72.64 | 0.05 | GZ |
| thymol | 41.47 | 0.03 | GZ |
| hexanoic acid | 73.08 | 0.01 | GZ |
| Ethyl methoxycinnamate | 23.36 | 0.06 | GZ |
| DEP | 52.19 | 0.07 | GZ |
| beta-asarone | 35.61 | 0.06 | GZ |
| Homocresol | 35.9 | 0.03 | GZ |
| (-)-Comphene | 34.98 | 0.04 | GZ |
| Peruviol | 29.61 | 0.06 | GZ |
| Iva | 62.17 | 0.01 | GZ |
| (R)-2-methylbutyric acid | 28.86 | 0.01 | GZ |
| Ethylcinnamate | 20.54 | 0.04 | GZ |
| chavicol | 44.19 | 0.02 | GZ |
| (4S)-1-methyl-4-(6-methylhepta-1,5-dien-2-yl)cyclohexene | 20.3 | 0.06 | GZ |
| Guasol | 51.6 | 0.02 | GZ |
| IPH | 36.05 | 0.01 | GZ |
| nonanoic acid | 40.51 | 0.02 | GZ |
| alpha cadinene | 18.73 | 0.08 | GZ |
| Syringaldehyde | 67.06 | 0.05 | GZ |
| (1S,4S)-7-isopropylidene-1,4-dimethyl-2,3,4,5,6,8-hexahydro-1H-azulene | 24.38 | 0.07 | GZ |
| naphthalene | 27.55 | 0.03 | GZ |
| Tolualdehydes | 44.18 | 0.02 | GZ |
| Isohomogenol | 32.61 | 0.04 | GZ |
| Benzenepropanol | 36.57 | 0.02 | GZ |
| CADINENE | 17.12 | 0.08 | GZ |
| T-Cadinol | 28.59 | 0.09 | GZ |
| calacorene | 16.2 | 0.08 | GZ |
| Benzyl acetate | 20.79 | 0.03 | GZ |
| Naphthalene, 1,2,3,4,4a,5,6,8a-octahydro-7-methyl-4-methylene-1-(1-methylethyl)-, (1alpha,4abeta,8aalpha)- | 20.21 | 0.08 | GZ |
| α-Cadinene | 14.76 | 0.08 | GZ |
| 2-Coumarate | 53.6 | 0.04 | GZ |
| o-cresol | 62.45 | 0.02 | GZ |
| acetic acid | 47.87 | 0 | GZ |
| taxifolin | 57.84 | 0.27 | GZ |
| heptanoic acid | 13.38 | 0.01 | GZ |
| tau-cadinol | 36.51 | 0.09 | GZ |
| (3R,4aR,8aR)-3-isopropenyl-5,8a-dimethyl-2,3,4,4a,7,8-hexahydro-1H-naphthalene | 23.86 | 0.08 | GZ |
| (1R,4aR,8aS)-1-isopropyl-7-methyl-4-methylene-2,3,4a,5,6,8a-hexahydro-1H-naphthalene | 21.35 | 0.08 | GZ |
| Stenol | 12.66 | 0.11 | GZ |
| 1,2-Benzenedicarboxylicacid, mono(2-ethyl) hexylester | 55.17 | 0.13 | GZ |
| WLN: QR BV1 | 24.15 | 0.03 | GZ |
| Tetradecanal | 12.36 | 0.05 | GZ |
| Papite | 31.04 | 0 | GZ |
| Clorius | 45.99 | 0.02 | GZ |
| α-muurolene | 15.64 | 0.08 | GZ |
| (1R,8aS)-4-isopropyl-1,6-dimethyl-1,2,3,7,8,8a-hexahydronaphthalene | 16.04 | 0.08 | GZ |
| 19435-97-3 | 33.04 | 0.09 | GZ |
| (1S,4R,4aR,8aR)-1-isopropyl-4,7-dimethyl-2,3,4,5,6,8a-hexahydro-1H-naphthalen-4a-ol | 62.54 | 0.09 | GZ |
| Isoamyl benzoate | 52.2 | 0.05 | GZ |
| Apple oil | 22.26 | 0.03 | GZ |
| ()-alpha-Funebrene | 49.41 | 0.1 | GZ |
| (Z)-calamenene | 17.75 | 0.08 | GZ |
| bergamotene (Z,.alpha.,cis) | 18.43 | 0.09 | GZ |
| Peroxyergosterol | 44.39 | 0.82 | GZ |
| Phenethyl acetate | 22.99 | 0.04 | GZ |
| (1R,3R,5R)-6,6-dimethyl-2-methylene-3-norpinanol | 51.38 | 0.06 | GZ |
| d-Piperitone | 48.75 | 0.03 | GZ |
| eremophilene | 34.6 | 0.08 | GZ |
| o-Anisic acid | 73.92 | 0.03 | GZ |
| (4aR,9aS)-2,9,9-trimethyl-5-methylene-4,4a,6,7,8,9a-hexahydro-3H-benzo[7]annulene | 46.05 | 0.08 | GZ |
| (1Z,4E,8E)-2,6,6,9-tetramethylcycloundeca-1,4,8-triene | 22.83 | 0.06 | GZ |
| caryophellene | 23.79 | 0.09 | GZ |
| Biosol | 44.42 | 0.03 | GZ |
| p-Methoxycinnamaldehyde | 59.64 | 0.04 | GZ |
