# Supplementary material for: Fuling-Guizhi Herb Pair in Coronary Heart Disease: Integrating Network Pharmacology and In Vivo Pharmacological Evaluation
Source: Evid Based Complement Alternat Med. 2020 May 17;2020:1489036. doi: 10.1155/2020/1489036 (PMC7251461; doi:10.1155/2020/1489036)
Supplement: Supplementary Materials — Supplementary Table S1: the detailed information of ingredients in FL and GZ. Supplementary Table S2: the detailed target information of compounds in FGHP. Supplementary Table S3: targets related to CHD. Supplementary Table S4: overlapping targets between FGHP and CHD. Supplementary Table S5: GO and pathway enrichment analysis by DAVID. [file 1489036.f1.zip › 1489036.f1/Supplementary Table S2. The detailed target information of compounds in FGHP .docx]

**Supplementary Table S2. The detailed target information of compounds in FGHP**

| Description | Symbol |
| --- | --- |
| DNA topoisomerase II alpha | TOP2A |
| Protein-tyrosine phosphatase 1B | PTPN1 |
| Prostaglandin E synthase | PTGES |
| TNF-alpha | TNF |
| Androgen Receptor | AR |
| Cytochrome P450 19A1 | CYP19A1 |
| Nitric oxide synthase, inducible | NOS2 |
| Carboxylesterase 2 | CES2 |
| LXR-alpha | NR1H3 |
| CD81 antigen | CD81 |
| Glucocorticoid receptor | NR3C1 |
| Protein farnesyltransferase | FNTA FNTB |
| 11-beta-hydroxysteroid dehydrogenase 1 | HSD11B1 |
| Protein-tyrosine phosphatase 1C | PTPN6 |
| G protein-coupled receptor 44 | PTGDR2 |
| Steroid 5-alpha-reductase 2 | SRD5A2 |
| Cysteinyl leukotriene receptor 1 | CYSLTR1 |
| Mineralocorticoid receptor | NR3C2 |
| DNA polymerase beta (by homology) | POLB |
| Prostanoid EP2 receptor (by homology) | PTGER2 |
| Dual specificity phosphatase Cdc25B | CDC25B |
| Testis-specific androgen-binding protein | SHBG |
| Vitamin D receptor | VDR |
| Corticosteroid binding globulin | SERPINA6 |
| Angiotensin-converting enzyme | ACE |
| Glucose-6-phosphate 1-dehydrogenase | G6PD |
| Cytochrome P450 51 (by homology) | CYP51A1 |
| Arachidonate 12-lipoxygenase | ALOX12 |
| Peroxisome proliferator-activated receptor alpha | PPARA |
| Peroxisome proliferator-activated receptor delta | PPARD |
| Nuclear receptor ROR-gamma | RORC |
| Receptor-type tyrosine-protein phosphatase F (LAR) | PTPRF |
| T-cell protein-tyrosine phosphatase | PTPN2 |
| Low molecular weight phosphotyrosine protein phosphatase | ACP1 |
| Aldo-keto reductase family 1 member B10 | AKR1B10 |
| 11-beta-hydroxysteroid dehydrogenase 2 | HSD11B2 |
| Prolyl endopeptidase | PREP |
| Phosphodiesterase 4D | PDE4D |
| Phospholipase A2 group 1B | PLA2G1B |
| Estrogen receptor alpha | ESR1 |
| Estrogen receptor beta | ESR2 |
| Peroxisome proliferator-activated receptor gamma | PPARG |
| Niemann-Pick C1-like protein 1 | NPC1L1 |
| Sigma opioid receptor | SIGMAR1 |
| Cytochrome P450 17A1 | CYP17A1 |
| RAS guanyl releasing protein 3 | RASGRP3 |
| Hydroxyacid oxidase 1 | HAO1 |
| Fatty acid binding protein epidermal | FABP5 |
| Fatty acid-binding protein, liver | FABP1 |
| Prostanoid EP4 receptor (by homology) | PTGER4 |
| Beta-secretase 1 | BACE1 |
| Delta opioid receptor | OPRD1 |
| Kappa Opioid receptor | OPRK1 |
| Matrix metalloproteinase 3 | MMP3 |
| Matrix metalloproteinase 1 | MMP1 |
| Matrix metalloproteinase 2 | MMP2 |
| Prostanoid FP receptor | PTGFR |
| SUMO-activating enzyme | SAE1 UBA2 |
| Fatty acid binding protein adipocyte | FABP4 |
| Fatty acid binding protein muscle | FABP3 |
| Protein-tyrosine phosphatase 2C | PTPN11 |
| Telomerase reverse transcriptase | TERT |
| Acyl-CoA desaturase | SCD |
| Protein kinase C eta | PRKCH |
| Dopamine transporter | SLC6A3 |
| Adenosine A3 receptor | ADORA3 |
| MAP kinase ERK1 | MAPK3 |
| DNA topoisomerase I | TOP1 |
| HMG-CoA reductase | HMGCR |
| Nuclear receptor ROR-alpha | RORA |
| Progesterone receptor | PGR |
| Dual specificity phosphatase Cdc25A | CDC25A |
| Cyclooxygenase-1 | PTGS1 |
| Free fatty acid receptor 1 | FFAR1 |
| Anandamide amidohydrolase | FAAH |
| Butyrylcholinesterase | BCHE |
| Prostanoid IP receptor | PTGIR |
| Muscarinic acetylcholine receptor M2 | CHRM2 |
| Norepinephrine transporter | SLC6A2 |
| Serotonin transporter | SLC6A4 |
| Cytochrome P450 2C19 | CYP2C19 |
| Prostanoid EP1 receptor (by homology) | PTGER1 |
| Cannabinoid receptor 1 (by homology) | CNR1 |
| Arachidonate 5-lipoxygenase | ALOX5 |
| Sterol regulatory element-binding protein 2 | SREBF2 |
| Cathepsin D | CTSD |
| Cyclooxygenase-2 | PTGS2 |
| Indoleamine 2,3-dioxygenase | IDO1 |
| Acyl coenzyme A:cholesterol acyltransferase | CES1 |
| Pregnane X receptor | NR1I2 |
| Estradiol 17-beta-dehydrogenase 3 | HSD17B3 |
| Prostanoid EP3 receptor | PTGER3 |
| Solute carrier family 22 member 6 (by homology) | SLC22A6 |
| Acetylcholinesterase | ACHE |
| Dual specificity phosphatase Cdc25C | CDC25C |
| Leukotriene B4 receptor 1 | LTB4R |
| Nuclear receptor subfamily 1 group I member 3 (by homology) | NR1I3 |
| Vanilloid receptor | TRPV1 |
| Bile acid receptor FXR | NR1H4 |
| G-protein coupled bile acid receptor 1 | GPBAR1 |
| 5-lipoxygenase activating protein | ALOX5AP |
| Liver glycogen phosphorylase | PYGL |
| p53-binding protein Mdm-2 | MDM2 |
| Isocitrate dehydrogenase [NADP] cytoplasmic | IDH1 |
| Cannabinoid receptor 2 (by homology) | CNR2 |
| Interleukin-6 receptor subunit beta | IL6ST |
| Metabotropic glutamate receptor 1 | GRM1 |
| HERG | KCNH2 |
| Sphingosine 1-phosphate receptor Edg-3 | S1PR3 |
| Sphingosine 1-phosphate receptor Edg-1 | S1PR1 |
| Orexin receptor 2 | HCRTR2 |
| Orexin receptor 1 | HCRTR1 |
| Adenosine A1 receptor | ADORA1 |
| Thrombin and coagulation factor X | F10 |
| Adenosine A2a receptor | ADORA2A |
| Serine/threonine-protein kinase PLK1 | PLK1 |
| Phosphodiesterase 10A (by homology) | PDE10A |
| Kinesin-like protein 1 | KIF11 |
| Mixed lineage kinase 7 | MAP3K20 |
| TGF-beta receptor type II | TGFBR2 |
| TGF-beta receptor type I | TGFBR1 |
| PI3-kinase p110-beta subunit | PIK3CB |
| Neurokinin 2 receptor | TACR2 |
| Hepatocyte growth factor receptor | MET |
| Vasopressin V1a receptor | AVPR1A |
| MAP kinase p38 alpha | MAPK14 |
| Corticotropin releasing factor receptor 1 (by homology) | CRHR1 |
| c-Jun N-terminal kinase 1 | MAPK8 |
| Aldo-keto-reductase family 1 member C3 | AKR1C3 |
| Glutamate NMDA receptor; GRIN1/GRIN2A | GRIN2A GRIN1 |
| Glutamate receptor ionotropic, AMPA 2 | GRIA2 |
| C-X-C chemokine receptor type 7 | ACKR3 |
| Beta amyloid A4 protein | APP |
| Intercellular adhesion molecule (ICAM-1), Integrin alpha-L/beta-2 | ITGAL ICAM1 ITGB2 |
| C5a anaphylatoxin chemotactic receptor | C5AR1 |
| Cytochrome P450 2C9 | CYP2C9 |
| Cytochrome P450 3A4 | CYP3A4 |
| Gamma-secretase | PSEN2 PSENEN NCSTN APH1A PSEN1 APH1B |
| c-Jun N-terminal kinase 3 | MAPK10 |
| c-Jun N-terminal kinase 2 | MAPK9 |
| Phosphodiesterase 2A | PDE2A |
| Serine/threonine-protein kinase mTOR | MTOR |
| PI3-kinase p110-alpha subunit | PIK3CA |
| Cathepsin L | CTSL |
| Geranylgeranyl transferase type I | PGGT1B FNTA |
| Tyrosine-protein kinase ITK/TSK | ITK |
| P2X purinoceptor 3 | P2RX3 |
| ALK tyrosine kinase receptor | ALK |
| CDC7/DBF4 (Cell division cycle 7-related protein kinase/Activator of S phase kinase) | CDC7 |
| Vascular endothelial growth factor receptor 2 | KDR |
| Macrophage colony stimulating factor receptor | CSF1R |
| Stem cell growth factor receptor | KIT |
| Muscarinic acetylcholine receptor M1 | CHRM1 |
| Cyclin-dependent kinase 1 | CDK1 |
| Melatonin receptor 1A | MTNR1A |
| Melatonin receptor 1B | MTNR1B |
| GABA-A receptor; alpha-3/beta-3/gamma-2 | GABRB3 GABRA3 GABRG2 |
| GABA-A receptor; alpha-1/beta-3/gamma-2 | GABRB3 GABRG2 GABRA1 |
| GABA-A receptor; alpha-5/beta-3/gamma-2 | GABRB3 GABRG2 GABRA5 |
| GABA-A receptor; alpha-2/beta-3/gamma-2 | GABRA2 GABRB3 GABRG2 |
| Monocarboxylate transporter 1 | SLC16A1 |
| Nerve growth factor receptor Trk-A | NTRK1 |
| Tyrosine-protein kinase JAK2 | JAK2 |
| Fatty acid synthase | FASN |
| Hematopoietic prostaglandin D synthase | HPGDS |
| Protein kinase C delta | PRKCD |
| Protein kinase C theta | PRKCQ |
| Smoothened homolog | SMO |
| EZH2/SUZ12/EED/RBBP7/RBBP4 | EZH2 |
| Proto-oncogene tyrosine-protein kinase MER | MERTK |
| Protein kinase C alpha | PRKCA |
| Thrombin | F2 |
| 3-beta-hydroxysteroid dehydrogenase/delta 5-->4-isomerase type II | HSD3B2 |
| Protein Mdm4 | MDM4 |
| Voltage-gated calcium channel alpha2/delta subunit 1 | CACNA2D1 |
| Glycogen synthase kinase-3 beta | GSK3B |
| Glycine receptor subunit alpha-1 | GLRA1 |
| Anti-estrogen binding site (AEBS) (by homology) | DHCR7 |
| LXR-beta | NR1H2 |
| Protein kinase C gamma | PRKCG |
| Protein kinase C beta | PRKCB |
| Protein kinase C epsilon | PRKCE |
| Sonic hedgehog protein (by homology) | SHH |
| Neurokinin 1 receptor | TACR1 |
| Calcium sensing receptor | CASR |
| Cyclin-dependent kinase 6 | CDK6 |
| Cyclin-dependent kinase 4 | CDK4 |
| Thromboxane A2 receptor | TBXA2R |
| Dopamine D2 receptor | DRD2 |
| C-C chemokine receptor type 1 | CCR1 |
| Interleukin-8 receptor A | CXCR1 |
| Carbonic anhydrase II | CA2 |
| Carbonic anhydrase VII | CA7 |
| Carbonic anhydrase I | CA1 |
| Carbonic anhydrase III | CA3 |
| Carbonic anhydrase VI | CA6 |
| Carbonic anhydrase XII | CA12 |
| Carbonic anhydrase IX | CA9 |
| Carbonic anhydrase IV | CA4 |
| Carbonic anhydrase VB | CA5B |
| Carbonic anhydrase VA | CA5A |
| P-glycoprotein 1 | ABCB1 |
| Matrix metalloproteinase 9 | MMP9 |
| Apoptosis regulator Bcl-2 | BCL2 |
| Matrix metalloproteinase 13 | MMP13 |
| Matrix metalloproteinase 12 | MMP12 |
| Placenta growth factor | PGF |
| Vascular endothelial growth factor A | VEGFA |
| Tyrosine-protein kinase SRC | SRC |
| Fibroblast growth factor receptor 1 | FGFR1 |
| Microtubule-associated protein tau | MAPT |
| DNA (cytosine-5)-methyltransferase 1 | DNMT1 |
| Dual-specificity tyrosine-phosphorylation regulated kinase 1A | DYRK1A |
| 6-phosphogluconate dehydrogenase | PGD |
| CMP-N-acetylneuraminate-beta-1,4-galactoside alpha-2,3-sialyltransferase | ST3GAL3 |
| Alpha-(1,3)-fucosyltransferase 7 | FUT7 |
| Matrix metalloproteinase 14 | MMP14 |
| Fucosyltransferase 4 | FUT4 |
| Signal transducer and activator of transcription 1-alpha/beta | STAT1 |
| Squalene monooxygenase (by homology) | SQLE |
| GABA-A receptor; alpha-1/beta-2/gamma-2 | GABRA1 GABRB2 GABRG2 |
| Hypoxia-inducible factor 1 alpha | HIF1A |
| Cytochrome P450 1B1 | CYP1B1 |
| Carbonic anhydrase XIII | CA13 |
| Kallikrein 1 | KLK1 |
| Kallikrein 2 | KLK2 |
| Multidrug resistance-associated protein 1 | ABCC1 |
| Estradiol 17-beta-dehydrogenase 1 | HSD17B1 |
| Carbonyl reductase [NADPH] 1 | CBR1 |
| Receptor protein-tyrosine kinase erbB-2 | ERBB2 |
| Vascular endothelial growth factor receptor 1 | FLT1 |
| Epidermal growth factor receptor erbB1 | EGFR |
| Taste receptor type 2 member 31 | TAS2R31 |
| ATP-binding cassette sub-family G member 2 | ABCG2 |
| Monoamine oxidase B | MAOB |
| Estradiol 17-beta-dehydrogenase 2 | HSD17B2 |
| Aldo-keto reductase family 1 member C2 | AKR1C2 |
| Endothelial PAS domain-containing protein 1 | EPAS1 |
| Trypsin I | PRSS1 |
| Chymotrypsin C | CTRC |
| NACHT, LRR and PYD domains-containing protein 3 | NLRP3 |
| Phospholipase A2 group IIA | PLA2G2A |
| Aldo-keto reductase family 1 member C1 | AKR1C1 |
| Inhibitor of nuclear factor kappa B kinase beta subunit | IKBKB |
| Sphingosine 1-phosphate receptor Edg-5 | S1PR2 |
| Cholecystokinin B receptor (by homology) | CCKBR |
| Type-1 angiotensin II receptor (by homology) | AGTR1 |
| Endothelin-converting enzyme 1 | ECE1 |
| Ephrin type-A receptor 2 | EPHA2 |
| Thromboxane-A synthase | TBXAS1 |
| Endothelin receptor ET-B | EDNRB |
| FK506-binding protein 1A | FKBP1A |
| Peptidyl-prolyl cis-trans isomerase FKBP5 | FKBP5 |
| Cytosolic phospholipase A2 | PLA2G4A |
| FK506 binding protein 4 | FKBP4 |
| Integrin alpha-V/beta-3 | ITGAV ITGB3 |
| Inosine-5'-monophosphate dehydrogenase 2 | IMPDH2 |
| Prostanoid DP receptor | PTGDR |
| Inosine-5'-monophosphate dehydrogenase 1 | IMPDH1 |
| Cytochrome P450 26A1 | CYP26A1 |
| Thymidylate synthase (by homology) | TYMS |
| Coagulation factor XI | F11 |
| AMP deaminase 2 | AMPD2 |
| Glutamate receptor ionotropic kainate 1 | GRIK1 |
| Glutamate receptor ionotropic kainate 2 | GRIK2 |
| Interleukin-6 | IL6 |
| Glutamine synthetase | GLUL |
| Thyroid hormone receptor alpha | THRA |
| Thyroid hormone receptor beta-1 | THRB |
| Squalene synthetase (by homology) | FDFT1 |
| UDP-glucuronosyltransferase 2B7 | UGT2B7 |
| Retinoid X receptor alpha | RXRA |
| Sodium/glucose cotransporter 2 | SLC5A2 |
| Metabotropic glutamate receptor 5 | GRM5 |
| Phospholipase A2 group V | PLA2G5 |
| Group X secretory phospholipase A2 | PLA2G10 |
| Plasminogen activator inhibitor-1 | SERPINE1 |
