# Supplementary material for: Fuling-Guizhi Herb Pair in Coronary Heart Disease: Integrating Network Pharmacology and In Vivo Pharmacological Evaluation
Source: Evid Based Complement Alternat Med. 2020 May 17;2020:1489036. doi: 10.1155/2020/1489036 (PMC7251461; doi:10.1155/2020/1489036)
Supplement: Supplementary Materials — Supplementary Table S1: the detailed information of ingredients in FL and GZ. Supplementary Table S2: the detailed target information of compounds in FGHP. Supplementary Table S3: targets related to CHD. Supplementary Table S4: overlapping targets between FGHP and CHD. Supplementary Table S5: GO and pathway enrichment analysis by DAVID. [file 1489036.f1.zip › 1489036.f1/Supplementary Table S3. Targets related to CHD .docx]

**Supplementary Table S3. Targets related to CHD**

| Symbol | Description |
| --- | --- |
| LDLR | low density lipoprotein receptor |
| PLA2G7 | phospholipase A2 group VII |
| APOB | apolipoprotein B |
| LPA | lipoprotein(a) |
| APOE | apolipoprotein E |
| MTHFR | methylenetetrahydrofolate reductase |
| TCF7L2 | transcription factor 7 like 2 |
| HNF1A | HNF1 homeobox A |
| ABCG5 | ATP binding cassette subfamily G member 5 |
| PON1 | paraoxonase 1 |
| ACE | angiotensin I converting enzyme |
| DNAH11 | dynein axonemal heavy chain 11 |
| ZC3HC1 | zinc finger C3HC-type containing 1 |
| MRAS | muscle RAS oncogene homolog |
| CX3CR1 | C-X3-C motif chemokine receptor 1 |
| MMP3 | matrix metallopeptidase 3 |
| IRS1 | insulin receptor substrate 1 |
| CD36 | CD36 molecule |
| VKORC1 | vitamin K epoxide reductase complex subunit 1 |
| KALRN | kalirin RhoGEF kinase |
| ESR2 | estrogen receptor 2 |
| PLAU | plasminogen activator, urokinase |
| MMP9 | matrix metallopeptidase 9 |
| MIR17HG | miR-17-92a-1 cluster host gene |
| MACO1 | macoilin 1 |
| CCDC92 | coiled-coil domain containing 92 |
| CCL2 | C-C motif chemokine ligand 2 |
| EDN1 | endothelin 1 |
| CCR5 | C-C motif chemokine receptor 5 (gene/pseudogene) |
| CD40LG | CD40 ligand |
| LPL | lipoprotein lipase |
| LOX | lysyl oxidase |
| LIPC | lipase C, hepatic type |
| ABCC9 | ATP binding cassette subfamily C member 9 |
| KCNJ8 | potassium voltage-gated channel subfamily J member 8 |
| PCSK9 | proprotein convertase subtilisin/kexin type 9 |
| CETP | cholesteryl ester transfer protein |
| ABCA1 | ATP binding cassette subfamily A member 1 |
| CDKN2B-AS1 | CDKN2B antisense RNA 1 |
| PHACTR1 | phosphatase and actin regulator 1 |
| ADAMTS7 | ADAM metallopeptidase with thrombospondin type 1 motif 7 |
| HMGCR | 3-hydroxy-3-methylglutaryl-CoA reductase |
| SCARB1 | scavenger receptor class B member 1 |
| ALDH2 | aldehyde dehydrogenase 2 family member |
| FADS1 | fatty acid desaturase 1 |
| ALOX5 | arachidonate 5-lipoxygenase |
| MTHFD1L | methylenetetrahydrofolate dehydrogenase (NADP+ dependent) 1 like |
| PLPP3 | phospholipid phosphatase 3 |
| AR | androgen receptor |
| FADS2 | fatty acid desaturase 2 |
| MIA3 | MIA SH3 domain ER export factor 3 |
| NR1H3 | nuclear receptor subfamily 1 group H member 3 |
| LIPA | lipase A, lysosomal acid type |
| SMAD3 | SMAD family member 3 |
| GALNT2 | polypeptide N-acetylgalactosaminyltransferase 2 |
| ADTRP | androgen dependent TFPI regulating protein |
| ZPR1 | ZPR1 zinc finger |
| PROCR | protein C receptor |
| SVEP1 | sushi, von Willebrand factor type A, EGF and pentraxin domain containing 1 |
| KCNJ11 | potassium voltage-gated channel subfamily J member 11 |
| UBE2Z | ubiquitin conjugating enzyme E2 Z |
| FTO | FTO, alpha-ketoglutarate dependent dioxygenase |
| SH2B3 | SH2B adaptor protein 3 |
| JCAD | junctional cadherin 5 associated |
| ABCG8 | ATP binding cassette subfamily G member 8 |
| BRAP | BRCA1 associated protein |
| SMARCA4 | SWI/SNF related, matrix associated, actin dependent regulator of chromatin, subfamily a, member 4 |
| ZBTB7C | zinc finger and BTB domain containing 7C |
| PPARG | peroxisome proliferator activated receptor gamma |
| GCKR | glucokinase regulator |
| SMG6 | SMG6, nonsense mediated mRNA decay factor |
| SIK3 | SIK family kinase 3 |
| ANKS1A | ankyrin repeat and sterile alpha motif domain containing 1A |
| GATA4 | GATA binding protein 4 |
| DOCK9 | dedicator of cytokinesis 9 |
| CUX2 | cut like homeobox 2 |
| FLT1 | fms related tyrosine kinase 1 |
| GRHL1 | grainyhead like transcription factor 1 |
| POLK | DNA polymerase kappa |
| MECOM | MDS1 and EVI1 complex locus |
| CARMIL1 | capping protein regulator and myosin 1 linker 1 |
| POLR3B | RNA polymerase III subunit B |
| ESR1 | estrogen receptor 1 |
| WDR12 | WD repeat domain 12 |
| BCAP29 | B cell receptor associated protein 29 |
| SLC12A9 | solute carrier family 12 member 9 |
| EEF1A2 | eukaryotic translation elongation factor 1 alpha 2 |
| CMSS1 | cms1 ribosomal small subunit homolog (yeast) |
| ZCCHC8 | zinc finger CCHC-type containing 8 |
| MAML3 | mastermind like transcriptional coactivator 3 |
| F7 | coagulation factor VII |
| FHIT | fragile histidine triad |
| GALNT7 | polypeptide N-acetylgalactosaminyltransferase 7 |
| SEMA5B | semaphorin 5B |
| CNNM2 | cyclin and CBS domain divalent metal cation transport mediator 2 |
| CDKAL1 | CDK5 regulatory subunit associated protein 1 like 1 |
| ULK4 | unc-51 like kinase 4 |
| CHDH | choline dehydrogenase |
| RNF130 | ring finger protein 130 |
| STK32B | serine/threonine kinase 32B |
| GABRB1 | gamma-aminobutyric acid type A receptor beta1 subunit |
| PLCG1 | phospholipase C gamma 1 |
| TP53BP1 | tumor protein p53 binding protein 1 |
| TRIO | trio Rho guanine nucleotide exchange factor |
| VEGFA | vascular endothelial growth factor A |
| ZNF165 | zinc finger protein 165 |
| SLC30A3 | solute carrier family 30 member 3 |
| MEF2A | myocyte enhancer factor 2A |
| BAZ1B | bromodomain adjacent to zinc finger domain 1B |
| ADIPOQ | adiponectin, C1Q and collagen domain containing |
| KCNQ1 | potassium voltage-gated channel subfamily Q member 1 |
| ITPR3 | inositol 1,4,5-trisphosphate receptor type 3 |
| TNF | tumor necrosis factor |
| MYL2 | myosin light chain 2 |
| PPP1R12B | protein phosphatase 1 regulatory subunit 12B |
| REN | renin |
| PLA2G2A | phospholipase A2 group IIA |
| PLA2G1B | phospholipase A2 group IB |
| ATXN2 | ataxin 2 |
| SCN2A | sodium voltage-gated channel alpha subunit 2 |
| SERPINE1 | serpin family E member 1 |
| SKIV2L | Ski2 like RNA helicase |
| SLC22A1 | solute carrier family 22 member 1 |
| NOTCH2 | notch 2 |
| NOS3 | nitric oxide synthase 3 |
| ITPK1 | inositol-tetrakisphosphate 1-kinase |
| KIAA0319 | KIAA0319 |
| TRAFD1 | TRAF-type zinc finger domain containing 1 |
| HP | haptoglobin |
| HNF4A | hepatocyte nuclear factor 4 alpha |
| HMOX1 | heme oxygenase 1 |
| FILIP1L | filamin A interacting protein 1 like |
| HFE | homeostatic iron regulator |
| CEP162 | centrosomal protein 162 |
| GSTT1 | glutathione S-transferase theta 1 |
| RPH3A | rabphilin 3A |
| SORCS3 | sortilin related VPS10 domain containing receptor 3 |
| SLC17A3 | solute carrier family 17 member 3 |
| IGF2BP2 | insulin like growth factor 2 mRNA binding protein 2 |
| TOMM40 | translocase of outer mitochondrial membrane 40 |
| IL18 | interleukin 18 |
| SLC17A4 | solute carrier family 17 member 4 |
| COL4A3BP | collagen type IV alpha 3 binding protein |
| IL6 | interleukin 6 |
| RAD50 | RAD50 double strand break repair protein |
| NUTF2 | nuclear transport factor 2 |
| IL1B | interleukin 1 beta |
| HCG9 | HLA complex group 9 |
| BTN3A3 | butyrophilin subfamily 3 member A3 |
| BTN2A2 | butyrophilin subfamily 2 member A2 |
| ASTN2 | astrotactin 2 |
| ACAD10 | acyl-CoA dehydrogenase family member 10 |
| DNAJC5B | DnaJ heat shock protein family (Hsp40) member C5 beta |
| HCG24 | HLA complex group 24 |
| HCG18 | HLA complex group 18 |
| HCG17 | HLA complex group 17 |
| CD14 | CD14 molecule |
| PLEKHA7 | pleckstrin homology domain containing A7 |
| APOC3 | apolipoprotein C3 |
| TMEM231 | transmembrane protein 231 |
| HLA-V | major histocompatibility complex, class I, V (pseudogene) |
| CR1L | complement C3b/C4b receptor 1 like |
| CHRDL1 | chordin like 1 |
| KLHL29 | kelch like family member 29 |
| CRP | C-reactive protein |
| CSK | C-terminal Src kinase |
| FRMD5 | FERM domain containing 5 |
| SRRM4 | serine/arginine repetitive matrix 4 |
| APOA1 | apolipoprotein A1 |
| DIAPH3 | diaphanous related formin 3 |
| CHRNA3 | cholinergic receptor nicotinic alpha 3 subunit |
| KCTD10 | potassium channel tetramerization domain containing 10 |
| NLRC5 | NLR family CARD domain containing 5 |
| TOMM5 | translocase of outer mitochondrial membrane 5 |
| HHIPL1 | HHIP like 1 |
| UBE2Q2P1 | ubiquitin conjugating enzyme E2 Q2 pseudogene 1 |
| FNDC1 | fibronectin type III domain containing 1 |
| AGTR1 | angiotensin II receptor type 1 |
| COL4A2 | collagen type IV alpha 2 chain |
| AGT | angiotensinogen |
| COL11A2 | collagen type XI alpha 2 chain |
| LHFPL3 | LHFPL tetraspan subfamily member 3 |
| NAA25 | N(alpha)-acetyltransferase 25, NatB auxiliary subunit |
| HECTD4 | HECT domain E3 ubiquitin protein ligase 4 |
| FAM114A1 | family with sequence similarity 114 member A1 |
| PLCD3 | phospholipase C delta 3 |
| DPEP3 | dipeptidase 3 |
| DPEP2 | dipeptidase 2 |
| GPSM3 | G protein signaling modulator 3 |
| AGAP1 | ArfGAP with GTPase domain, ankyrin repeat and PH domain 1 |
| CYP17A1 | cytochrome P450 family 17 subfamily A member 1 |
| ZNF831 | zinc finger protein 831 |
| ZFHX3 | zinc finger homeobox 3 |
| ATP2B1 | ATPase plasma membrane Ca2+ transporting 1 |
| THADA | THADA, armadillo repeat containing |
| ABL1 | ABL proto-oncogene 1, non-receptor tyrosine kinase |
| SLC30A8 | solute carrier family 30 member 8 |
| GLT1D1 | glycosyltransferase 1 domain containing 1 |
| SUGP1 | SURP and G-patch domain containing 1 |
| TTC39B | tetratricopeptide repeat domain 39B |
| DNM2 | dynamin 2 |
| APOA5 | apolipoprotein A5 |
| ZNRD1ASP | zinc ribbon domain containing 1 antisense, pseudogene |
| KIF12 | kinesin family member 12 |
| CYBA | cytochrome b-245 alpha chain |
| TTC41P | tetratricopeptide repeat domain 41, pseudogene |
| JAZF1 | JAZF zinc finger 1 |
| CACNB2 | calcium voltage-gated channel auxiliary subunit beta 2 |
| GLCCI1 | glucocorticoid induced 1 |
| LINC00240 | long intergenic non-protein coding RNA 240 |
| CABCOCO1 | ciliary associated calcium binding coiled-coil 1 |
| ZBED9 | zinc finger BED-type containing 9 |
| ADAMTS9-AS2 | ADAMTS9 antisense RNA 2 |
| CYP2C19 | cytochrome P450 family 2 subfamily C member 19 |
| BTD | biotinidase |
| TARID | TCF21 antisense RNA inducing promoter demethylation |
| LINC01339 | long intergenic non-protein coding RNA 1339 |
| ZSCAN31 | zinc finger and SCAN domain containing 31 |
| APOA4 | apolipoprotein A4 |
| ITGB3 | integrin subunit beta 3 |
| TLR4 | toll like receptor 4 |
| MTRR | 5-methyltetrahydrofolate-homocysteine methyltransferase reductase |
| FGB | fibrinogen beta chain |
| VWF | von Willebrand factor |
| PLA2G6 | phospholipase A2 group VI |
| NKX2-5 | NK2 homeobox 5 |
| VDR | vitamin D receptor |
| GSTM1 | glutathione S-transferase mu 1 |
| SORT1 | sortilin 1 |
| NFKB1 | nuclear factor kappa B subunit 1 |
| AGER | advanced glycosylation end-product specific receptor |
| CXCL12 | C-X-C motif chemokine ligand 12 |
| P2RY12 | purinergic receptor P2Y12 |
| LTA | lymphotoxin alpha |
| OLR1 | oxidized low density lipoprotein receptor 1 |
| IL1RN | interleukin 1 receptor antagonist |
| IL1A | interleukin 1 alpha |
| CAD | carbamoyl-phosphate synthetase 2, aspartate transcarbamylase, and dihydroorotase |
| USF1 | upstream transcription factor 1 |
| CFH | complement factor H |
| PON2 | paraoxonase 2 |
| MPO | myeloperoxidase |
| ALB | albumin |
| ALOX5AP | arachidonate 5-lipoxygenase activating protein |
| HPGDS | hematopoietic prostaglandin D synthase |
| IL10 | interleukin 10 |
| SELP | selectin P |
| CDKN2B | cyclin dependent kinase inhibitor 2B |
| CDKN2A | cyclin dependent kinase inhibitor 2A |
| F2 | coagulation factor II, thrombin |
| ADD1 | adducin 1 |
| RETN | resistin |
| PTGS2 | prostaglandin-endoperoxide synthase 2 |
| KIF6 | kinesin family member 6 |
| DECR1 | 2,4-dienoyl-CoA reductase 1 |
| KL | klotho |
| OR10A4 | olfactory receptor family 10 subfamily A member 4 |
| PPARA | peroxisome proliferator activated receptor alpha |
| CCR2 | C-C motif chemokine receptor 2 |
| NR3C1 | nuclear receptor subfamily 3 group C member 1 |
| MTR | 5-methyltetrahydrofolate-homocysteine methyltransferase |
| GSTK1 | glutathione S-transferase kappa 1 |
| IL6R | interleukin 6 receptor |
| THBD | thrombomodulin |
| AMPD1 | adenosine monophosphate deaminase 1 |
| SLCO6A1 | solute carrier organic anion transporter family member 6A1 |
| GNB3 | G protein subunit beta 3 |
| LIPG | lipase G, endothelial type |
| CXCL16 | C-X-C motif chemokine ligand 16 |
| NPC1L1 | NPC1 like intracellular cholesterol transporter 1 |
| TBX5 | T-box 5 |
| ABCC6 | ATP binding cassette subfamily C member 6 |
| TNFRSF11B | TNF receptor superfamily member 11b |
| C20orf181 | chromosome 20 open reading frame 181 |
| CYP1A1 | cytochrome P450 family 1 subfamily A member 1 |
| ATM | ATM serine/threonine kinase |
| UCP2 | uncoupling protein 2 |
| SELE | selectin E |
| CCL5 | C-C motif chemokine ligand 5 |
| PON3 | paraoxonase 3 |
| KNG1 | kininogen 1 |
| PECAM1 | platelet and endothelial cell adhesion molecule 1 |
| PRH2 | proline rich protein HaeIII subfamily 2 |
| MIR126 | microRNA 126 |
| PLG | plasminogen |
| ABCB1 | ATP binding cassette subfamily B member 1 |
| PLA2G10 | phospholipase A2 group X |
| LMNA | lamin A/C |
| GJA4 | gap junction protein alpha 4 |
| GPR162 | G protein-coupled receptor 162 |
| GATA6 | GATA binding protein 6 |
| GATA2 | GATA binding protein 2 |
| HIF1A | hypoxia inducible factor 1 subunit alpha |
| ANGPTL3 | angiopoietin like 3 |
| F3 | coagulation factor III, tissue factor |
| ANGPTL4 | angiopoietin like 4 |
| TCF21 | transcription factor 21 |
| CYP2J2 | cytochrome P450 family 2 subfamily J member 2 |
| CPB2 | carboxypeptidase B2 |
| HAND1 | heart and neural crest derivatives expressed 1 |
| F5 | coagulation factor V |
| FABP2 | fatty acid binding protein 2 |
| WDTC1 | WD and tetratricopeptide repeats 1 |
| CITED2 | Cbp/p300 interacting transactivator with Glu/Asp rich carboxy-terminal domain 2 |
| MIR146A | microRNA 146a |
| SIRT1 | sirtuin 1 |
| APOA2 | apolipoprotein A2 |
| CYP2B6 | cytochrome P450 family 2 subfamily B member 6 |
| GP1BA | glycoprotein Ib platelet subunit alpha |
| KIF28P | kinesin family member 28, pseudogene |
| ALOX15 | arachidonate 15-lipoxygenase |
| MMP2 | matrix metallopeptidase 2 |
| MTTP | microsomal triglyceride transfer protein |
| UGT1A1 | UDP glucuronosyltransferase family 1 member A1 |
| NPY | neuropeptide Y |
| SLC6A4 | solute carrier family 6 member 4 |
| FLNA | filamin A |
| NOS2 | nitric oxide synthase 2 |
| SELPLG | selectin P ligand |
| ICAM1 | intercellular adhesion molecule 1 |
| SELL | selectin L |
| TFPI | tissue factor pathway inhibitor |
| TGFB1 | transforming growth factor beta 1 |
| TP53 | tumor protein p53 |
| MIR499A | microRNA 499a |
| TNFRSF1B | TNF receptor superfamily member 1B |
| MUSK | muscle associated receptor tyrosine kinase |
| FLAD1 | flavin adenine dinucleotide synthetase 1 |
| GP6 | glycoprotein VI platelet |
| YWHAZ | tyrosine 3-monooxygenase/tryptophan 5-monooxygenase activation protein zeta |
| ZIC3 | Zic family member 3 |
| CBS | cystathionine-beta-synthase |
| EPHX2 | epoxide hydrolase 2 |
| ABO | ABO, alpha 1-3-N-acetylgalactosaminyltransferase and alpha 1-3-galactosyltransferase |
| HSPD1 | heat shock protein family D (Hsp60) member 1 |
| RAPSN | receptor associated protein of the synapse |
| LGALS2 | galectin 2 |
| PTGS1 | prostaglandin-endoperoxide synthase 1 |
| LEP | leptin |
| NLRP3 | NLR family pyrin domain containing 3 |
| NAMPT | nicotinamide phosphoribosyltransferase |
| TBX20 | T-box 20 |
| APOC1 | apolipoprotein C1 |
| CST3 | cystatin C |
| MMRN1 | multimerin 1 |
| CXCR4 | C-X-C motif chemokine receptor 4 |
| ITGA2B | integrin subunit alpha 2b |
| MBL2 | mannose binding lectin 2 |
| CD59 | CD59 molecule (CD59 blood group) |
| CYP2C8 | cytochrome P450 family 2 subfamily C member 8 |
| KIF2C | kinesin family member 2C |
| PLB1 | phospholipase B1 |
| SHBG | sex hormone binding globulin |
| PPIA | peptidylprolyl isomerase A |
| GHRL | ghrelin and obestatin prepropeptide |
| ARL15 | ADP ribosylation factor like GTPase 15 |
| SEMA6A | semaphorin 6A |
| FSD1 | fibronectin type III and SPRY domain containing 1 |
| PRH1 | proline rich protein HaeIII subfamily 1 |
| ACE2 | angiotensin I converting enzyme 2 |
| TRIB3 | tribbles pseudokinase 3 |
| GMCL2 | germ cell-less, spermatogenesis associated 2 |
| GMCL1 | germ cell-less, spermatogenesis associated 1 |
| SCN5A | sodium voltage-gated channel alpha subunit 5 |
| QRSL1 | glutaminyl-tRNA synthase (glutamine-hydrolyzing)-like 1 |
| RBP4 | retinol binding protein 4 |
| COX8A | cytochrome c oxidase subunit 8A |
| CBSL | cystathionine-beta-synthase like |
| PROC | protein C, inactivator of coagulation factors Va and VIIIa |
| APOM | apolipoprotein M |
| ROS1 | ROS proto-oncogene 1, receptor tyrosine kinase |
| DAB2IP | DAB2 interacting protein |
| JPH3 | junctophilin 3 |
| SLCO1B1 | solute carrier organic anion transporter family member 1B1 |
| NEXN | nexilin F-actin binding protein |
| HPSE | heparanase |
| IL33 | interleukin 33 |
| UTS2 | urotensin 2 |
| PSRC1 | proline and serine rich coiled-coil 1 |
| CUBN | cubilin |
| CFDP1 | craniofacial development protein 1 |
| KLF2 | Kruppel like factor 2 |
| NTN1 | netrin 1 |
| BCAR1 | BCAR1, Cas family scaffold protein |
| ABCG1 | ATP binding cassette subfamily G member 1 |
| NOS1AP | nitric oxide synthase 1 adaptor protein |
| NR1H4 | nuclear receptor subfamily 1 group H member 4 |
| TMEM170A | transmembrane protein 170A |
| F2RL3 | F2R like thrombin or trypsin receptor 3 |
| TRIB1 | tribbles pseudokinase 1 |
| ATXN2L | ataxin 2 like |
| CD93 | CD93 molecule |
| XRCC1 | X-ray repair cross complementing 1 |
| TERC | telomerase RNA component |
| EHMT1 | euchromatic histone lysine methyltransferase 1 |
| MIR223 | microRNA 223 |
| ADIPOR1 | adiponectin receptor 1 |
| TBC1D9 | TBC1 domain family member 9 |
| SOD1 | superoxide dismutase 1 |
| INSIG2 | insulin induced gene 2 |
| HSPA14 | heat shock protein family A (Hsp70) member 14 |
| SETD2 | SET domain containing 2 |
| THBS1 | thrombospondin 1 |
| AVSD1 | atrioventricular septal defect 1 |
| FSD1L | fibronectin type III and SPRY domain containing 1 like |
| HEY2 | hes related family bHLH transcription factor with YRPW motif 2 |
| LPAL2 | lipoprotein(a) like 2, pseudogene |
| MIR155 | microRNA 155 |
| GCA | grancalcin |
| POU2F3 | POU class 2 homeobox 3 |
| MIR21 | microRNA 21 |
| SLC22A3 | solute carrier family 22 member 3 |
| PPBP | pro-platelet basic protein |
| BDNF | brain derived neurotrophic factor |
| HSD11B1 | hydroxysteroid 11-beta dehydrogenase 1 |
| FBN1 | fibrillin 1 |
| FABP4 | fatty acid binding protein 4 |
| MMP12 | matrix metallopeptidase 12 |
| HSPA4 | heat shock protein family A (Hsp70) member 4 |
| CASR | calcium sensing receptor |
| FAS | Fas cell surface death receptor |
| CASQ2 | calsequestrin 2 |
| CD28 | CD28 molecule |
| FCGR2A | Fc fragment of IgG receptor IIa |
| APLNR | apelin receptor |
| MGP | matrix Gla protein |
| HLA-DQB1 | major histocompatibility complex, class II, DQ beta 1 |
| FCGR3B | Fc fragment of IgG receptor IIIb |
| PCSK1 | proprotein convertase subtilisin/kexin type 1 |
| MIF | macrophage migration inhibitory factor |
| FCGR3A | Fc fragment of IgG receptor IIIa |
| INSIG1 | insulin induced gene 1 |
| INS | insulin |
| F2R | coagulation factor II thrombin receptor |
| IGF1 | insulin like growth factor 1 |
| IGF2 | insulin like growth factor 2 |
| BMPR2 | bone morphogenetic protein receptor type 2 |
| MYH6 | myosin heavy chain 6 |
| ECE1 | endothelin converting enzyme 1 |
| BCL2 | BCL2, apoptosis regulator |
| CELSR2 | cadherin EGF LAG seven-pass G-type receptor 2 |
| EDNRA | endothelin receptor type A |
| NODAL | nodal growth differentiation factor |
| CXCL8 | C-X-C motif chemokine ligand 8 |
| BHMT | betaine--homocysteine S-methyltransferase |
| ETS1 | ETS proto-oncogene 1, transcription factor |
| EPRS | glutamyl-prolyl-tRNA synthetase |
| COX1 | cytochrome c oxidase subunit I |
| TNC | tenascin C |
| COX2 | cytochrome c oxidase subunit II |
| IL15 | interleukin 15 |
| C3 | complement C3 |
| FOXO3 | forkhead box O3 |
| PPARD | peroxisome proliferator activated receptor delta |
| ADRB2 | adrenoceptor beta 2 |
| ADRB1 | adrenoceptor beta 1 |
| ADRA2B | adrenoceptor alpha 2B |
| CYP3A4 | cytochrome P450 family 3 subfamily A member 4 |
| ACAT2 | acetyl-CoA acetyltransferase 2 |
| GCLC | glutamate-cysteine ligase catalytic subunit |
| ADH1C | alcohol dehydrogenase 1C (class I), gamma polypeptide |
| GJA5 | gap junction protein alpha 5 |
| CNR1 | cannabinoid receptor 1 |
| PLTP | phospholipid transfer protein |
| ACTC1 | actin, alpha, cardiac muscle 1 |
| LCAT | lecithin-cholesterol acyltransferase |
| ACTA2 | actin, alpha 2, smooth muscle, aorta |
| GC | GC, vitamin D binding protein |
| LEPR | leptin receptor |
| CLU | clusterin |
| CES1 | carboxylesterase 1 |
| PIK3CG | phosphatidylinositol-4,5-bisphosphate 3-kinase catalytic subunit gamma |
| PGF | placental growth factor |
| FN1 | fibronectin 1 |
| CYP11B2 | cytochrome P450 family 11 subfamily B member 2 |
| FSHMD1A | facioscapulohumeral muscular dystrophy 1A |
| CRELD1 | cysteine rich with EGF like domains 1 |
| HPSE2 | heparanase 2 (inactive) |
| CSF2 | colony stimulating factor 2 |
| DEFA1 | defensin alpha 1 |
| KIF9 | kinesin family member 9 |
| MKL1 | megakaryoblastic leukemia (translocation) 1 |
| HEATR6 | HEAT repeat containing 6 |
| CRH | corticotropin releasing hormone |
| CYP1A2 | cytochrome P450 family 1 subfamily A member 2 |
| CPS1 | carbamoyl-phosphate synthase 1 |
| NOX5 | NADPH oxidase 5 |
| SLC52A2 | solute carrier family 52 member 2 |
| KIDINS220 | kinase D interacting substrate 220 |
| CYP3A5 | cytochrome P450 family 3 subfamily A member 5 |
| CSF3 | colony stimulating factor 3 |
| DNAH8 | dynein axonemal heavy chain 8 |
| NQO1 | NAD(P)H quinone dehydrogenase 1 |
| ROBO3 | roundabout guidance receptor 3 |
| CYP4A11 | cytochrome P450 family 4 subfamily A member 11 |
| DEFA3 | defensin alpha 3 |
| DSCAM | DS cell adhesion molecule |
| CYP2D6 | cytochrome P450 family 2 subfamily D member 6 |
| CYP2C9 | cytochrome P450 family 2 subfamily C member 9 |
| SENP2 | SUMO specific peptidase 2 |
| RNF213 | ring finger protein 213 |
| CYP7A1 | cytochrome P450 family 7 subfamily A member 1 |
| NDST4 | N-deacetylase and N-sulfotransferase 4 |
| CYBB | cytochrome b-245 beta chain |
| HOMEZ | homeobox and leucine zipper encoding |
| DTNA | dystrobrevin alpha |
| GREM1 | gremlin 1, DAN family BMP antagonist |
| ZBTB21 | zinc finger and BTB domain containing 21 |
| IRX4 | iroquois homeobox 4 |
| FOXP3 | forkhead box P3 |
| ASCC1 | activating signal cointegrator 1 complex subunit 1 |
| MLXIPL | MLX interacting protein like |
| FXN | frataxin |
| FOS | Fos proto-oncogene, AP-1 transcription factor subunit |
| FOLH1 | folate hydrolase 1 |
| WNT16 | Wnt family member 16 |
| ISYNA1 | inositol-3-phosphate synthase 1 |
| FOXO1 | forkhead box O1 |
| EHD3 | EH domain containing 3 |
| FUT3 | fucosyltransferase 3 (Lewis blood group) |
| SENP1 | SUMO specific peptidase 1 |
| FOXP1 | forkhead box P1 |
| B3GAT1 | beta-1,3-glucuronyltransferase 1 |
| PALD1 | phosphatase domain containing, paladin 1 |
| C5AR2 | complement component 5a receptor 2 |
| PDCD4 | programmed cell death 4 |
| GAPDH | glyceraldehyde-3-phosphate dehydrogenase |
| GALNT3 | polypeptide N-acetylgalactosaminyltransferase 3 |
| GAD1 | glutamate decarboxylase 1 |
| NXT1 | nuclear transport factor 2 like export factor 1 |
| MYLIP | myosin regulatory light chain interacting protein |
| GABPA | GA binding protein transcription factor subunit alpha |
| CES1P1 | carboxylesterase 1 pseudogene 1 |
| FEN1 | flap structure-specific endonuclease 1 |
| EPHX1 | epoxide hydrolase 1 |
| SELENOS | selenoprotein S |
| MESP1 | mesoderm posterior bHLH transcription factor 1 |
| MARK2 | microtubule affinity regulating kinase 2 |
| ELANE | elastase, neutrophil expressed |
| CFC1 | cripto, FRL-1, cryptic family 1 |
| SLC2A9 | solute carrier family 2 member 9 |
| EGR1 | early growth response 1 |
| PRDM10 | PR/SET domain 10 |
| EGFR | epidermal growth factor receptor |
| AS3MT | arsenite methyltransferase |
| ETS2 | ETS proto-oncogene 2, transcription factor |
| EXTL3 | exostosin like glycosyltransferase 3 |
| PARL | presenilin associated rhomboid like |
| HDL3 | Huntington-like neurodegenerative disorder 2 |
| RIPK4 | receptor interacting serine/threonine kinase 4 |
| CCHCR1 | coiled-coil alpha-helical rod protein 1 |
| FCGR2B | Fc fragment of IgG receptor IIb |
| FCAR | Fc fragment of IgA receptor |
| BCOR | BCL6 corepressor |
| FBN2 | fibrillin 2 |
| F13A1 | coagulation factor XIII A chain |
| RNLS | renalase, FAD dependent amine oxidase |
| F12 | coagulation factor XII |
| F8 | coagulation factor VIII |
| NLN | neurolysin |
| ARSA | arylsulfatase A |
| SUMO4 | small ubiquitin-like modifier 4 |
| ARMS2 | age-related maculopathy susceptibility 2 |
| ANXA1 | annexin A1 |
| ANGPT2 | angiopoietin 2 |
| CIMT | Carotid intimal medial thickness |
| MIRLET7I | microRNA let-7i |
| AKT1 | AKT serine/threonine kinase 1 |
| MIR145 | microRNA 145 |
| AHSG | alpha 2-HS glycoprotein |
| MIR150 | microRNA 150 |
| MIR17 | microRNA 17 |
| MIR197 | microRNA 197 |
| ANXA2 | annexin A2 |
| APC | APC, WNT signaling pathway regulator |
| SEMA3D | semaphorin 3D |
| ARNTL | aryl hydrocarbon receptor nuclear translocator like |
| AQP5 | aquaporin 5 |
| TAS2R50 | taste 2 receptor member 50 |
| APOD | apolipoprotein D |
| SERPINA9 | serpin family A member 9 |
| HCAR2 | hydroxycarboxylic acid receptor 2 |
| NANOS3 | nanos C2HC-type zinc finger 3 |
| APOC4 | apolipoprotein C4 |
| IRF2BP2 | interferon regulatory factor 2 binding protein 2 |
| APOC2 | apolipoprotein C2 |
| PEAR1 | platelet endothelial aggregation receptor 1 |
| MIR19B1 | microRNA 19b-1 |
| MIR20A | microRNA 20a |
| MIR206 | microRNA 206 |
| ZGLP1 | zinc finger, GATA-like protein 1 |
| MIR365A | microRNA 365a |
| ADH1A | alcohol dehydrogenase 1A (class I), alpha polypeptide |
| ACVR1 | activin A receptor type 1 |
| HLP | hyperkeratosis lenticularis perstans |
| MIR2909 | microRNA 2909 |
| ACP1 | acid phosphatase 1 |
| MIR4513 | microRNA 4513 |
| PGR-AS1 | PGR antisense RNA 1 |
| ACHE | acetylcholinesterase (Cartwright blood group) |
| TP53COR1 | tumor protein p53 pathway corepressor 1 |
| THRA1/BTR | uncharacterized LOC105371807 |
| ADH1B | alcohol dehydrogenase 1B (class I), beta polypeptide |
| MIR486-1 | microRNA 486-1 |
| MIR208A | microRNA 208a |
| AGTR2 | angiotensin II receptor type 2 |
| MIR214 | microRNA 214 |
| ADRB3 | adrenoceptor beta 3 |
| MIR224 | microRNA 224 |
| MIR23A | microRNA 23a |
| MIR31 | microRNA 31 |
| MIR34A | microRNA 34a |
| ADRA2A | adrenoceptor alpha 2A |
| OR13G1 | olfactory receptor family 13 subfamily G member 1 |
| MIR361 | microRNA 361 |
| ADORA3 | adenosine A3 receptor |
| RN7SL263P | RNA, 7SL, cytoplasmic 263, pseudogene |
| ARSD | arylsulfatase D |
| CPE | carboxypeptidase E |
| CHIT1 | chitinase 1 |
| ARID5B | AT-rich interaction domain 5B |
| CHI3L1 | chitinase 3 like 1 |
| CCL21 | C-C motif chemokine ligand 21 |
| CDH13 | cadherin 13 |
| CDK1 | cyclin dependent kinase 1 |
| CD44 | CD44 molecule (Indian blood group) |
| CNDP1 | carnosine dipeptidase 1 |
| BUD13 | BUD13 homolog |
| CD40 | CD40 molecule |
| CD34 | CD34 molecule |
| CD86 | CD86 molecule |
| ESYT3 | extended synaptotagmin 3 |
| ST6GALNAC5 | ST6 N-acetylgalactosaminide alpha-2,6-sialyltransferase 5 |
| ADIPOR2 | adiponectin receptor 2 |
| CP | ceruloplasmin |
| CORT | cortistatin |
| STN1 | STN1, CST complex subunit |
| COMT | catechol-O-methyltransferase |
| COL6A1 | collagen type VI alpha 1 chain |
| COL4A1 | collagen type IV alpha 1 chain |
| SP6 | Sp6 transcription factor |
| ADAM33 | ADAM metallopeptidase domain 33 |
| COL3A1 | collagen type III alpha 1 chain |
| ACKR2 | atypical chemokine receptor 2 |
| CCR7 | C-C motif chemokine receptor 7 |
| MCFD2 | multiple coagulation factor deficiency 2 |
| TBL1Y | transducin beta like 1 Y-linked |
| CAV1 | caveolin 1 |
| PWAR1 | Prader Willi/Angelman region RNA 1 |
| IL23R | interleukin 23 receptor |
| BCHE | butyrylcholinesterase |
| AVP | arginine vasopressin |
| SLC7A13 | solute carrier family 7 member 13 |
| ATP5PF | ATP synthase peripheral stalk subunit F6 |
| ATP4B | ATPase H+/K+ transporting subunit beta |
| ZNF627 | zinc finger protein 627 |
| DAND5 | DAN domain BMP antagonist family member 5 |
| SERPINC1 | serpin family C member 1 |
| ASGR1 | asialoglycoprotein receptor 1 |
| DLEU7 | deleted in lymphocytic leukemia, 7 |
| SERPINA12 | serpin family A member 12 |
| BCL2A1 | BCL2 related protein A1 |
| OPN4 | opsin 4 |
| CASP1 | caspase 1 |
| SLC25A20 | solute carrier family 25 member 20 |
| CACNA1C | calcium voltage-gated channel subunit alpha1 C |
| CA2 | carbonic anhydrase 2 |
| KLF5 | Kruppel like factor 5 |
| CMTM5 | CKLF like MARVEL transmembrane domain containing 5 |
| BMP4 | bone morphogenetic protein 4 |
| EARS2 | glutamyl-tRNA synthetase 2, mitochondrial |
| BGLAP | bone gamma-carboxyglutamate protein |
| OSR1 | odd-skipped related transciption factor 1 |
| KLF14 | Kruppel like factor 14 |
| ASD1 | atrial septal defect 1 |
| FGF21 | fibroblast growth factor 21 |
| NPPC | natriuretic peptide C |
| NNMT | nicotinamide N-methyltransferase |
| NHS | NHS actin remodeling regulator |
| TCN2 | transcobalamin 2 |
| NFKBIA | NFKB inhibitor alpha |
| TFAP2B | transcription factor AP-2 beta |
| NR2F2 | nuclear receptor subfamily 2 group F member 2 |
| NFE2L2 | nuclear factor, erythroid 2 like 2 |
| NEUROD1 | neuronal differentiation 1 |
| LEFTY2 | left-right determination factor 2 |
| TGFBR2 | transforming growth factor beta receptor 2 |
| NDUFS2 | NADH:ubiquinone oxidoreductase core subunit S2 |
| NCAM1 | neural cell adhesion molecule 1 |
| TBX3 | T-box 3 |
| NOS1 | nitric oxide synthase 1 |
| TBX1 | T-box 1 |
| NOTCH1 | notch 1 |
| SOD2 | superoxide dismutase 2 |
| SOD3 | superoxide dismutase 3 |
| SOX9 | SRY-box 9 |
| SPINK1 | serine peptidase inhibitor, Kazal type 1 |
| SPP1 | secreted phosphoprotein 1 |
| SREBF1 | sterol regulatory element binding transcription factor 1 |
| SREBF2 | sterol regulatory element binding transcription factor 2 |
| ST2 | suppression of tumorigenicity 2 |
| ST14 | suppression of tumorigenicity 14 |
| STK11 | serine/threonine kinase 11 |
| TAC1 | tachykinin precursor 1 |
| THBS4 | thrombospondin 4 |
| THRA | thyroid hormone receptor alpha |
| TIMP1 | TIMP metallopeptidase inhibitor 1 |
| WRN | Werner syndrome RecQ like helicase |
| MMP1 | matrix metallopeptidase 1 |
| NR3C2 | nuclear receptor subfamily 3 group C member 2 |
| MEFV | MEFV, pyrin innate immunity regulator |
| LRP8 | LDL receptor related protein 8 |
| MEF2C | myocyte enhancer factor 2C |
| ST8SIA4 | ST8 alpha-N-acetyl-neuraminide alpha-2,8-sialyltransferase 4 |
| ADAM11 | ADAM metallopeptidase domain 11 |
| GHS | Goldenhar syndrome |
| MAF | MAF bZIP transcription factor |
| FGF23 | fibroblast growth factor 23 |
| KMT2D | lysine methyltransferase 2D |
| WRB | tryptophan rich basic protein |
| MMP7 | matrix metallopeptidase 7 |
| MMP8 | matrix metallopeptidase 8 |
| MYBPC3 | myosin binding protein C, cardiac |
| TNFAIP3 | TNF alpha induced protein 3 |
| MYB | MYB proto-oncogene, transcription factor |
| TNNT1 | troponin T1, slow skeletal type |
| TRNA | tRNA |
| TRAF5 | TNF receptor associated factor 5 |
| TRPC3 | transient receptor potential cation channel subfamily C member 3 |
| TNFSF4 | TNF superfamily member 4 |
| SUMO1 | small ubiquitin-like modifier 1 |
| MT1B | metallothionein 1B |
| MMP14 | matrix metallopeptidase 14 |
| MMP13 | matrix metallopeptidase 13 |
| MIA | MIA SH3 domain containing |
| POU5F1 | POU class 5 homeobox 1 |
| RAC1 | Rac family small GTPase 1 |
| MOK | MOK protein kinase |
| PLGLB2 | plasminogen-like B2 |
| RBL2 | RB transcriptional corepressor like 2 |
| PLAT | plasminogen activator, tissue type |
| PLA2G5 | phospholipase A2 group V |
| RENBP | renin binding protein |
| RFC1 | replication factor C subunit 1 |
| PITX2 | paired like homeodomain 2 |
| RPA1 | replication protein A1 |
| RPN1 | ribophorin I |
| RXRA | retinoid X receptor alpha |
| PTX3 | pentraxin 3 |
| PTPRC | protein tyrosine phosphatase, receptor type C |
| PTPN1 | protein tyrosine phosphatase, non-receptor type 1 |
| POLD1 | DNA polymerase delta 1, catalytic subunit |
| PRKAA1 | protein kinase AMP-activated catalytic subunit alpha 1 |
| PRKCE | protein kinase C epsilon |
| MAPK3 | mitogen-activated protein kinase 3 |
| MAPK8 | mitogen-activated protein kinase 8 |
| PRKY | protein kinase Y-linked (pseudogene) |
| PNN | pinin, desmosome associated protein |
| PROS1 | protein S |
| PSMD9 | proteasome 26S subunit, non-ATPase 9 |
| TAS2R38 | taste 2 receptor member 38 |
| PTH | parathyroid hormone |
| PLGLB1 | plasminogen-like B1 |
| RYR2 | ryanodine receptor 2 |
| RYR3 | ryanodine receptor 3 |
| S100A9 | S100 calcium binding protein A9 |
| SERPINB2 | serpin family B member 2 |
| SFRP4 | secreted frizzled related protein 4 |
| SRSF1 | serine and arginine rich splicing factor 1 |
| SRSF2 | serine and arginine rich splicing factor 2 |
| SH3BGR | SH3 domain binding glutamate rich protein |
| PCSK6 | proprotein convertase subtilisin/kexin type 6 |
| P2RY1 | purinergic receptor P2Y1 |
| SLC2A3 | solute carrier family 2 member 3 |
| OPRM1 | opioid receptor mu 1 |
| SLC19A1 | solute carrier family 19 member 1 |
| NTRK3 | neurotrophic receptor tyrosine kinase 3 |
| NTRK2 | neurotrophic receptor tyrosine kinase 2 |
| PCDH8 | protocadherin 8 |
| PCNA | proliferating cell nuclear antigen |
| PCYT1A | phosphate cytidylyltransferase 1, choline, alpha |
| S100A12 | S100 calcium binding protein A12 |
| SCD | stearoyl-CoA desaturase |
| PF4V1 | platelet factor 4 variant 1 |
| SERPINF1 | serpin family F member 1 |
| SLC26A4 | solute carrier family 26 member 4 |
| ENPP1 | ectonucleotide pyrophosphatase/phosphodiesterase 1 |
| CCL18 | C-C motif chemokine ligand 18 |
| CCL19 | C-C motif chemokine ligand 19 |
| PDGFA | platelet derived growth factor subunit A |
| CCL22 | C-C motif chemokine ligand 22 |
| CXCL5 | C-X-C motif chemokine ligand 5 |
| CX3CL1 | C-X3-C motif chemokine ligand 1 |
| SMARCA2 | SWI/SNF related, matrix associated, actin dependent regulator of chromatin, subfamily a, member 2 |
| DLC1 | DLC1 Rho GTPase activating protein |
| NPFFR2 | neuropeptide FF receptor 2 |
| HMGA1 | high mobility group AT-hook 1 |
| SUB1 | SUB1 homolog, transcriptional regulator |
| HLA-DRB5 | major histocompatibility complex, class II, DR beta 5 |
| TMED1 | transmembrane p24 trafficking protein 1 |
| ADAMTS13 | ADAM metallopeptidase with thrombospondin type 1 motif 13 |
| HNRNPUL1 | heterogeneous nuclear ribonucleoprotein U like 1 |
| PTPRT | protein tyrosine phosphatase, receptor type T |
| CAPN10 | calpain 10 |
| HLA-DRB1 | major histocompatibility complex, class II, DR beta 1 |
| SLC2A6 | solute carrier family 2 member 6 |
| HAS2 | hyaluronan synthase 2 |
| HSPA1A | heat shock protein family A (Hsp70) member 1A |
| CYSLTR1 | cysteinyl leukotriene receptor 1 |
| NES | nestin |
| YAP1 | Yes associated protein 1 |
| IRF8 | interferon regulatory factor 8 |
| EIF3M | eukaryotic translation initiation factor 3 subunit M |
| CAP1 | cyclase associated actin cytoskeleton regulatory protein 1 |
| ANP32B | acidic nuclear phosphoprotein 32 family member B |
| HTC2 | hypertrichosis 2 (generalized, congenital) |
| MRPL28 | mitochondrial ribosomal protein L28 |
| HSPG2 | heparan sulfate proteoglycan 2 |
| LEFTY1 | left-right determination factor 1 |
| HSPA8 | heat shock protein family A (Hsp70) member 8 |
| CXCR6 | C-X-C motif chemokine receptor 6 |
| EBP | EBP, cholestenol delta-isomerase |
| HAL | histidine ammonia-lyase |
| GUK1 | guanylate kinase 1 |
| GSTM2 | glutathione S-transferase mu 2 |
| ZFPM2 | zinc finger protein, FOG family member 2 |
| ANGPTL2 | angiopoietin like 2 |
| GDF1 | growth differentiation factor 1 |
| DDAH2 | dimethylarginine dimethylaminohydrolase 2 |
| DDAH1 | dimethylarginine dimethylaminohydrolase 1 |
| SMUG1 | single-strand-selective monofunctional uracil-DNA glycosylase 1 |
| BHMT2 | betaine--homocysteine S-methyltransferase 2 |
| GCK | glucokinase |
| GCH1 | GTP cyclohydrolase 1 |
| POC1A | POC1 centriolar protein A |
| GCG | glucagon |
| TES | testin LIM domain protein |
| GCLM | glutamate-cysteine ligase modifier subunit |
| GLP1R | glucagon like peptide 1 receptor |
| GLUL | glutamate-ammonia ligase |
| GRIK4 | glutamate ionotropic receptor kainate type subunit 4 |
| GPX1 | glutathione peroxidase 1 |
| SCAP | SREBF chaperone |
| GPT | glutamic--pyruvic transaminase |
| PALLD | palladin, cytoskeletal associated protein |
| GRK4 | G protein-coupled receptor kinase 4 |
| TAB2 | TGF-beta activated kinase 1 (MAP3K7) binding protein 2 |
| GPER1 | G protein-coupled estrogen receptor 1 |
| GPD1L | glycerol-3-phosphate dehydrogenase 1 like |
| ABRAXAS2 | abraxas 2, BRISC complex subunit |
| GOT2 | glutamic-oxaloacetic transaminase 2 |
| GNGT2 | G protein subunit gamma transducin 2 |
| PLA2G2D | phospholipase A2 group IID |
| MKKS | McKusick-Kaufman syndrome |
| KAT2B | lysine acetyltransferase 2B |
| NR1I2 | nuclear receptor subfamily 1 group I member 2 |
| ARHGEF7 | Rho guanine nucleotide exchange factor 7 |
| LMAN1 | lectin, mannose binding 1 |
| SELENBP1 | selenium binding protein 1 |
| HAP1 | huntingtin associated protein 1 |
| LCN2 | lipocalin 2 |
| LDB2 | LIM domain binding 2 |
| TBX18 | T-box 18 |
| FCGR2C | Fc fragment of IgG receptor IIc (gene/pseudogene) |
| IL1RL1 | interleukin 1 receptor like 1 |
| SLC33A1 | solute carrier family 33 member 1 |
| PROM1 | prominin 1 |
| CREG1 | cellular repressor of E1A stimulated genes 1 |
| TNFRSF11A | TNF receptor superfamily member 11a |
| USP9Y | ubiquitin specific peptidase 9 Y-linked |
| SMAD7 | SMAD family member 7 |
| SMAD1 | SMAD family member 1 |
| LTC4S | leukotriene C4 synthase |
| SRPX | sushi repeat containing protein X-linked |
| SOAT2 | sterol O-acyltransferase 2 |
| MADD | MAP kinase activating death domain |
| LTBP1 | latent transforming growth factor beta binding protein 1 |
| IRS2 | insulin receptor substrate 2 |
| VAMP8 | vesicle associated membrane protein 8 |
| LRP6 | LDL receptor related protein 6 |
| TNFSF13 | TNF superfamily member 13 |
| NREP | neuronal regeneration related protein |
| KLK1 | kallikrein 1 |
| KDR | kinase insert domain receptor |
| IL15RA | interleukin 15 receptor subunit alpha |
| IL18BP | interleukin 18 binding protein |
| IL7 | interleukin 7 |
| IL5 | interleukin 5 |
| IL4 | interleukin 4 |
| IL2 | interleukin 2 |
| MPZL2 | myelin protein zero like 2 |
| IL1RAP | interleukin 1 receptor accessory protein |
| IGFBP3 | insulin like growth factor binding protein 3 |
| ZMPSTE24 | zinc metallopeptidase STE24 |
| AKR1A1 | aldo-keto reductase family 1 member A1 |
| IGFBP1 | insulin like growth factor binding protein 1 |
| IL16 | interleukin 16 |
| IL17A | interleukin 17A |
| ELMO1 | engulfment and cell motility 1 |
| KCNA3 | potassium voltage-gated channel subfamily A member 3 |
| ITGAM | integrin subunit alpha M |
| ITGA2 | integrin subunit alpha 2 |
| ISL1 | ISL LIM homeobox 1 |
| QKI | QKI, KH domain containing RNA binding |
| ADAMTS1 | ADAM metallopeptidase with thrombospondin type 1 motif 1 |
| LITAF | lipopolysaccharide induced TNF factor |
| IRF6 | interferon regulatory factor 6 |
| CLOCK | clock circadian regulator |
| INSR | insulin receptor |
| HDAC9 | histone deacetylase 9 |
| PCLAF | PCNA clamp associated factor |
| IFNG | interferon gamma |
