# Supplementary material for: Fuling-Guizhi Herb Pair in Coronary Heart Disease: Integrating Network Pharmacology and In Vivo Pharmacological Evaluation
Source: Evid Based Complement Alternat Med. 2020 May 17;2020:1489036. doi: 10.1155/2020/1489036 (PMC7251461; doi:10.1155/2020/1489036)
Supplement: Supplementary Materials — Supplementary Table S1: the detailed information of ingredients in FL and GZ. Supplementary Table S2: the detailed target information of compounds in FGHP. Supplementary Table S3: targets related to CHD. Supplementary Table S4: overlapping targets between FGHP and CHD. Supplementary Table S5: GO and pathway enrichment analysis by DAVID. [file 1489036.f1.zip › 1489036.f1/Supplementary Table S4. Overlapping targets between FGHP and CHD.docx]

**Supplementary Table S4. Overlapping targets between FGHP and CHD**

| Description | Symbol |
| --- | --- |
| phospholipase A2 group X(PLA2G10) | PLA2G10 |
| placental growth factor(PGF) | PGF |
| adenosine A3 receptor(ADORA3) | ADORA3 |
| transforming growth factor beta receptor 2(TGFBR2) | TGFBR2 |
| 3-hydroxy-3-methylglutaryl-CoA reductase(HMGCR) | HMGCR |
| vascular endothelial growth factor A(VEGFA) | VEGFA |
| vitamin D (1,25- dihydroxyvitamin D3) receptor(VDR) | VDR |
| hydroxysteroid 11-beta dehydrogenase 1(HSD11B1) | HSD11B1 |
| angiotensin I converting enzyme(ACE) | ACE |
| fms related tyrosine kinase 1(FLT1) | FLT1 |
| thyroid hormone receptor, alpha(THRA) | THRA |
| ATP binding cassette subfamily B member 1(ABCB1) | ABCB1 |
| stearoyl-CoA desaturase(SCD) | SCD |
| endothelin converting enzyme 1(ECE1) | ECE1 |
| kallikrein 1(KLK1) | KLK1 |
| NPC1 like intracellular cholesterol transporter 1(NPC1L1) | NPC1L1 |
| sex hormone binding globulin(SHBG) | SHBG |
| estrogen receptor 1(ESR1) | ESR1 |
| peroxisome proliferator activated receptor gamma(PPARG) | PPARG |
| serpin family E member 1(SERPINE1) | SERPINE1 |
| phospholipase A2 group IB(PLA2G1B) | PLA2G1B |
| prostaglandin-endoperoxide synthase 2(PTGS2) | PTGS2 |
| peroxisome proliferator activated receptor delta(PPARD) | PPARD |
| prostaglandin-endoperoxide synthase 1(PTGS1) | PTGS1 |
| peroxisome proliferator activated receptor alpha(PPARA) | PPARA |
| estrogen receptor 2(ESR2) | ESR2 |
| mitogen-activated protein kinase 8(MAPK8) | MAPK8 |
| acetylcholinesterase (Cartwright blood group)(ACHE) | ACHE |
| mitogen-activated protein kinase 3(MAPK3) | MAPK3 |
| phospholipase A2 group IIA(PLA2G2A) | PLA2G2A |
| phospholipase A2 group V(PLA2G5) | PLA2G5 |
| solute carrier family 6 member 4(SLC6A4) | SLC6A4 |
| cannabinoid receptor 1(CNR1) | CNR1 |
| angiotensin II receptor type 1(AGTR1) | AGTR1 |
| interleukin 6(IL6) | IL6 |
| acid phosphatase 1, soluble(ACP1) | ACP1 |
| protein kinase C epsilon(PRKCE) | PRKCE |
| kinase insert domain receptor(KDR) | KDR |
| cysteinyl leukotriene receptor 1(CYSLTR1) | CYSLTR1 |
| cytochrome P450 family 3 subfamily A member 4(CYP3A4) | CYP3A4 |
| retinoid X receptor alpha(RXRA) | RXRA |
| hypoxia inducible factor 1 alpha subunit(HIF1A) | HIF1A |
| matrix metallopeptidase 13(MMP13) | MMP13 |
| matrix metallopeptidase 14(MMP14) | MMP14 |
| protein tyrosine phosphatase, non-receptor type 1(PTPN1) | PTPN1 |
| matrix metallopeptidase 12(MMP12) | MMP12 |
| sterol regulatory element binding transcription factor 2(SREBF2) | SREBF2 |
| epidermal growth factor receptor(EGFR) | EGFR |
| carbonic anhydrase 2(CA2) | CA2 |
| nuclear receptor subfamily 1 group H member 4(NR1H4) | NR1H4 |
| hematopoietic prostaglandin D synthase(HPGDS) | HPGDS |
| cytochrome P450 family 17 subfamily A member 1(CYP17A1) | CYP17A1 |
| tumor necrosis factor(TNF) | TNF |
| carboxylesterase 1(CES1) | CES1 |
| fatty acid binding protein 4(FABP4) | FABP4 |
| nitric oxide synthase 2(NOS2) | NOS2 |
| NLR family pyrin domain containing 3(NLRP3) | NLRP3 |
| cyclin dependent kinase 1(CDK1) | CDK1 |
| butyrylcholinesterase(BCHE) | BCHE |
| matrix metallopeptidase 9(MMP9) | MMP9 |
| matrix metallopeptidase 2(MMP2) | MMP2 |
| matrix metallopeptidase 1(MMP1) | MMP1 |
| coagulation factor II, thrombin(F2) | F2 |
| BCL2, apoptosis regulator(BCL2) | BCL2 |
| nuclear receptor subfamily 1 group I member 2(NR1I2) | NR1I2 |
| nuclear receptor subfamily 1 group H member 3(NR1H3) | NR1H3 |
| matrix metallopeptidase 3(MMP3) | MMP3 |
| cytochrome P450 family 2 subfamily C member 19(CYP2C19) | CYP2C19 |
| glutamate-ammonia ligase(GLUL) | GLUL |
| cytochrome P450 family 2 subfamily C member 9(CYP2C9) | CYP2C9 |
| androgen receptor(AR) | AR |
| calcium sensing receptor(CASR) | CASR |
| arachidonate 5-lipoxygenase(ALOX5) | ALOX5 |
| nuclear receptor subfamily 3 group C member 2(NR3C2) | NR3C2 |
| nuclear receptor subfamily 3 group C member 1(NR3C1) | NR3C1 |
| arachidonate 5-lipoxygenase activating protein(ALOX5AP) | ALOX5AP |
