# Supplementary material for: Fuling-Guizhi Herb Pair in Coronary Heart Disease: Integrating Network Pharmacology and In Vivo Pharmacological Evaluation
Source: Evid Based Complement Alternat Med. 2020 May 17;2020:1489036. doi: 10.1155/2020/1489036 (PMC7251461; doi:10.1155/2020/1489036)
Supplement: Supplementary Materials — Supplementary Table S1: the detailed information of ingredients in FL and GZ. Supplementary Table S2: the detailed target information of compounds in FGHP. Supplementary Table S3: targets related to CHD. Supplementary Table S4: overlapping targets between FGHP and CHD. Supplementary Table S5: GO and pathway enrichment analysis by DAVID. [file 1489036.f1.zip › 1489036.f1/Supplementary Table S5. GO and Pathway Enrichment Analysis by DAVID database .docx]

**Supplementary Table S5. GO and Pathway Enrichment Analysis by DAVID database**

| Category | Term | *P* Value |
| --- | --- | --- |
| GOTERM_MF_DIRECT | GO:0003707~steroid hormone receptor activity | 1.2E-10 |
| GOTERM_MF_DIRECT | GO:0008270~zinc ion binding | 6.23E-10 |
| GOTERM_MF_DIRECT | GO:0004879~RNA polymerase II transcription factor activity, ligand-activated sequence-specific DNA binding | 0.00000017 |
| GOTERM_BP_DIRECT | GO:0030574~collagen catabolic process | 0.000000318 |
| GOTERM_CC_DIRECT | GO:0031012~extracellular matrix | 0.000000344 |
| GOTERM_BP_DIRECT | GO:0071222~cellular response to lipopolysaccharide | 0.000000562 |
| GOTERM_MF_DIRECT | GO:0004222~metalloendopeptidase activity | 0.00000173 |
| GOTERM_CC_DIRECT | GO:0090575~RNA polymerase II transcription factor complex | 0.00000297 |
| GOTERM_BP_DIRECT | GO:0007566~embryo implantation | 0.00000651 |
| GOTERM_BP_DIRECT | GO:0030522~intracellular receptor signaling pathway | 0.00000735 |
| GOTERM_BP_DIRECT | GO:0006351~transcription, DNA-templated | 0.0000164 |
| GOTERM_BP_DIRECT | GO:0043401~steroid hormone mediated signaling pathway | 0.0000214 |
| GOTERM_MF_DIRECT | GO:0008144~drug binding | 0.0000215 |
| GOTERM_BP_DIRECT | GO:0045766~positive regulation of angiogenesis | 0.0000541 |
| GOTERM_BP_DIRECT | GO:0045944~positive regulation of transcription from RNA polymerase II promoter | 0.0000652 |
| GOTERM_BP_DIRECT | GO:0071456~cellular response to hypoxia | 0.0000702 |
| GOTERM_MF_DIRECT | GO:0043565~sequence-specific DNA binding | 0.0000721 |
| GOTERM_BP_DIRECT | GO:0010887~negative regulation of cholesterol storage | 0.000158 |
| GOTERM_MF_DIRECT | GO:0004887~thyroid hormone receptor activity | 0.000324 |
| GOTERM_BP_DIRECT | GO:0018107~peptidyl-threonine phosphorylation | 0.000491 |
| GOTERM_BP_DIRECT | GO:2000188~regulation of cholesterol homeostasis | 0.000549 |
| GOTERM_BP_DIRECT | GO:0043066~negative regulation of apoptotic process | 0.000662 |
| GOTERM_MF_DIRECT | GO:0001077~transcriptional activator activity, RNA polymerase II core promoter proximal region sequence-specific binding | 0.00069 |
| GOTERM_BP_DIRECT | GO:0043406~positive regulation of MAP kinase activity | 0.000713 |
| GOTERM_BP_DIRECT | GO:0042593~glucose homeostasis | 0.000743 |
| GOTERM_CC_DIRECT | GO:0043235~receptor complex | 0.000819 |
| GOTERM_BP_DIRECT | GO:0030949~positive regulation of vascular endothelial growth factor receptor signaling pathway | 0.001163628 |
| GOTERM_BP_DIRECT | GO:0051384~response to glucocorticoid | 0.001163628 |
| GOTERM_CC_DIRECT | GO:0005615~extracellular space | 0.00125694 |
| GOTERM_BP_DIRECT | GO:0048546~digestive tract morphogenesis | 0.001695256 |
| GOTERM_MF_DIRECT | GO:0005496~steroid binding | 0.001742494 |
| GOTERM_BP_DIRECT | GO:0014068~positive regulation of phosphatidylinositol 3-kinase signaling | 0.001841535 |
| GOTERM_BP_DIRECT | GO:0030307~positive regulation of cell growth | 0.002077028 |
| GOTERM_BP_DIRECT | GO:0001934~positive regulation of protein phosphorylation | 0.002201469 |
| GOTERM_BP_DIRECT | GO:0018105~peptidyl-serine phosphorylation | 0.002212815 |
| GOTERM_MF_DIRECT | GO:0020037~heme binding | 0.003228931 |
| GOTERM_BP_DIRECT | GO:0033138~positive regulation of peptidyl-serine phosphorylation | 0.00336506 |
| GOTERM_BP_DIRECT | GO:0051091~positive regulation of sequence-specific DNA binding transcription factor activity | 0.004444523 |
| GOTERM_BP_DIRECT | GO:0050482~arachidonic acid secretion | 0.004751441 |
| GOTERM_BP_DIRECT | GO:0001666~response to hypoxia | 0.005487364 |
| GOTERM_BP_DIRECT | GO:0045893~positive regulation of transcription, DNA-templated | 0.006410233 |
| GOTERM_CC_DIRECT | GO:0009897~external side of plasma membrane | 0.006466691 |
| GOTERM_BP_DIRECT | GO:0010634~positive regulation of epithelial cell migration | 0.006810576 |
| GOTERM_BP_DIRECT | GO:0035987~endodermal cell differentiation | 0.007378161 |
| GOTERM_CC_DIRECT | GO:0045121~membrane raft | 0.008042499 |
| GOTERM_BP_DIRECT | GO:0045994~positive regulation of translational initiation by iron | 0.010362205 |
| GOTERM_BP_DIRECT | GO:0002540~leukotriene production involved in inflammatory response | 0.010362205 |
| GOTERM_BP_DIRECT | GO:0019371~cyclooxygenase pathway | 0.010362205 |
| GOTERM_BP_DIRECT | GO:0060571~morphogenesis of an epithelial fold | 0.010362205 |
| GOTERM_MF_DIRECT | GO:0046982~protein heterodimerization activity | 0.010479009 |
| GOTERM_BP_DIRECT | GO:0008217~regulation of blood pressure | 0.011209952 |
| GOTERM_MF_DIRECT | GO:0004666~prostaglandin-endoperoxide synthase activity | 0.011501063 |
| GOTERM_MF_DIRECT | GO:0003990~acetylcholinesterase activity | 0.011501063 |
| GOTERM_MF_DIRECT | GO:0038052~RNA polymerase II transcription factor activity, estrogen-activated sequence-specific DNA binding | 0.011501063 |
| GOTERM_MF_DIRECT | GO:0005509~calcium ion binding | 0.011712924 |
| GOTERM_BP_DIRECT | GO:0001503~ossification | 0.012644365 |
| GOTERM_BP_DIRECT | GO:0000122~negative regulation of transcription from RNA polymerase II promoter | 0.013290097 |
| GOTERM_BP_DIRECT | GO:0006954~inflammatory response | 0.013653929 |
| GOTERM_MF_DIRECT | GO:0001046~core promoter sequence-specific DNA binding | 0.013678676 |
| GOTERM_BP_DIRECT | GO:0010871~negative regulation of receptor biosynthetic process | 0.015503598 |
| GOTERM_BP_DIRECT | GO:0060745~mammary gland branching involved in pregnancy | 0.015503598 |
| GOTERM_BP_DIRECT | GO:1900086~positive regulation of peptidyl-tyrosine autophosphorylation | 0.015503598 |
| GOTERM_BP_DIRECT | GO:0031622~positive regulation of fever generation | 0.015503598 |
| GOTERM_BP_DIRECT | GO:0055098~response to low-density lipoprotein particle | 0.015503598 |
| GOTERM_BP_DIRECT | GO:0006919~activation of cysteine-type endopeptidase activity involved in apoptotic process | 0.016558032 |
| GOTERM_MF_DIRECT | GO:0070644~vitamin D response element binding | 0.017202632 |
| GOTERM_MF_DIRECT | GO:0034056~estrogen response element binding | 0.017202632 |
| GOTERM_BP_DIRECT | GO:0071230~cellular response to amino acid stimulus | 0.018250213 |
| GOTERM_MF_DIRECT | GO:0000978~RNA polymerase II core promoter proximal region sequence-specific DNA binding | 0.018477156 |
| GOTERM_BP_DIRECT | GO:0038033~positive regulation of endothelial cell chemotaxis by VEGF-activated vascular endothelial growth factor receptor signaling pathway | 0.02570763 |
| GOTERM_BP_DIRECT | GO:0010891~negative regulation of sequestering of triglyceride | 0.02570763 |
| GOTERM_BP_DIRECT | GO:0060664~epithelial cell proliferation involved in salivary gland morphogenesis | 0.02570763 |
| GOTERM_BP_DIRECT | GO:0050927~positive regulation of positive chemotaxis | 0.02570763 |
| GOTERM_BP_DIRECT | GO:0038166~angiotensin-activated signaling pathway | 0.02570763 |
| GOTERM_BP_DIRECT | GO:0043410~positive regulation of MAPK cascade | 0.027729503 |
| GOTERM_CC_DIRECT | GO:0000790~nuclear chromatin | 0.028167137 |
| GOTERM_BP_DIRECT | GO:0042448~progesterone metabolic process | 0.030770532 |
| GOTERM_BP_DIRECT | GO:0060687~regulation of branching involved in prostate gland morphogenesis | 0.030770532 |
| GOTERM_BP_DIRECT | GO:0045630~positive regulation of T-helper 2 cell differentiation | 0.030770532 |
| GOTERM_BP_DIRECT | GO:0045861~negative regulation of proteolysis | 0.030770532 |
| GOTERM_BP_DIRECT | GO:1902894~negative regulation of pri-miRNA transcription from RNA polymerase II promoter | 0.030770532 |
| GOTERM_BP_DIRECT | GO:0050728~negative regulation of inflammatory response | 0.03088558 |
| GOTERM_BP_DIRECT | GO:0043583~ear development | 0.035807535 |
| GOTERM_BP_DIRECT | GO:0035357~peroxisome proliferator activated receptor signaling pathway | 0.035807535 |
| GOTERM_BP_DIRECT | GO:0010745~negative regulation of macrophage derived foam cell differentiation | 0.035807535 |
| GOTERM_BP_DIRECT | GO:0032230~positive regulation of synaptic transmission, GABAergic | 0.035807535 |
| GOTERM_MF_DIRECT | GO:0004252~serine-type endopeptidase activity | 0.038304212 |
| GOTERM_MF_DIRECT | GO:0005506~iron ion binding | 0.039806613 |
| GOTERM_BP_DIRECT | GO:0050790~regulation of catalytic activity | 0.040818767 |
| GOTERM_BP_DIRECT | GO:0010831~positive regulation of myotube differentiation | 0.040818767 |
| GOTERM_BP_DIRECT | GO:0019370~leukotriene biosynthetic process | 0.040818767 |
| GOTERM_CC_DIRECT | GO:0005641~nuclear envelope lumen | 0.041263937 |
| GOTERM_BP_DIRECT | GO:0010468~regulation of gene expression | 0.044814877 |
| GOTERM_MF_DIRECT | GO:0004601~peroxidase activity | 0.045228119 |
| GOTERM_BP_DIRECT | GO:0032461~positive regulation of protein oligomerization | 0.04580436 |
| GOTERM_BP_DIRECT | GO:0050730~regulation of peptidyl-tyrosine phosphorylation | 0.04580436 |
| KEGG_PATHWAY | cfa05200:Pathways in cancer | 4.60E-09 |
| KEGG_PATHWAY | cfa04066:HIF-1 signaling pathway | 2.50E-06 |
| KEGG_PATHWAY | cfa03320:PPAR signaling pathway | 3.22E-06 |
| KEGG_PATHWAY | cfa05219:Bladder cancer | 2.98E-05 |
| KEGG_PATHWAY | cfa04668:TNF signaling pathway | 3.48E-05 |
| KEGG_PATHWAY | cfa05142:Chagas disease (American trypanosomiasis) | 3.71E-05 |
| KEGG_PATHWAY | cfa00590:Arachidonic acid metabolism | 2.06E-04 |
| KEGG_PATHWAY | cfa05205:Proteoglycans in cancer | 3.33E-04 |
| KEGG_PATHWAY | cfa05133:Pertussis | 3.68E-04 |
| KEGG_PATHWAY | cfa04931:Insulin resistance | 4.17E-04 |
| KEGG_PATHWAY | cfa04014:Ras signaling pathway | 7.01E-04 |
| KEGG_PATHWAY | cfa05323:Rheumatoid arthritis | 8.17E-04 |
| KEGG_PATHWAY | cfa04621:NOD-like receptor signaling pathway | 9.44E-04 |
| KEGG_PATHWAY | cfa05160:Hepatitis C | 9.78E-04 |
| KEGG_PATHWAY | cfa05206:MicroRNAs in cancer | 0.001499063 |
| KEGG_PATHWAY | cfa04915:Estrogen signaling pathway | 0.001661989 |
| KEGG_PATHWAY | cfa05202:Transcriptional misregulation in cancer | 0.002363212 |
| KEGG_PATHWAY | cfa04510:Focal adhesion | 0.002466423 |
| KEGG_PATHWAY | cfa05212:Pancreatic cancer | 0.00255931 |
| KEGG_PATHWAY | cfa05145:Toxoplasmosis | 0.002679837 |
| KEGG_PATHWAY | cfa04976:Bile secretion | 0.002864382 |
| KEGG_PATHWAY | cfa05152:Tuberculosis | 0.003250514 |
| KEGG_PATHWAY | cfa04917:Prolactin signaling pathway | 0.00392755 |
| KEGG_PATHWAY | cfa04071:Sphingolipid signaling pathway | 0.004551545 |
| KEGG_PATHWAY | cfa04912:GnRH signaling pathway | 0.006517473 |
| KEGG_PATHWAY | cfa05161:Hepatitis B | 0.008883206 |
| KEGG_PATHWAY | cfa04930:Type II diabetes mellitus | 0.009100437 |
| KEGG_PATHWAY | cfa04151:PI3K-Akt signaling pathway | 0.010338301 |
| KEGG_PATHWAY | cfa04932:Non-alcoholic fatty liver disease (NAFLD) | 0.010516382 |
| KEGG_PATHWAY | cfa04015:Rap1 signaling pathway | 0.010557643 |
| KEGG_PATHWAY | cfa04370:VEGF signaling pathway | 0.015949244 |
| KEGG_PATHWAY | cfa05210:Colorectal cancer | 0.017434434 |
| KEGG_PATHWAY | cfa04726:Serotonergic synapse | 0.017625268 |
| KEGG_PATHWAY | cfa04614:Renin-angiotensin system | 0.017991984 |
| KEGG_PATHWAY | cfa04919:Thyroid hormone signaling pathway | 0.018698752 |
| KEGG_PATHWAY | cfa04270:Vascular smooth muscle contraction | 0.020384058 |
| KEGG_PATHWAY | cfa05140:Leishmaniasis | 0.020630036 |
| KEGG_PATHWAY | cfa00591:Linoleic acid metabolism | 0.022716007 |
| KEGG_PATHWAY | cfa04920:Adipocytokine signaling pathway | 0.025989008 |
| KEGG_PATHWAY | cfa04520:Adherens junction | 0.025989008 |
| KEGG_PATHWAY | cfa04380:Osteoclast differentiation | 0.027352489 |
| KEGG_PATHWAY | cfa05216:Thyroid cancer | 0.029713093 |
| KEGG_PATHWAY | cfa04068:FoxO signaling pathway | 0.03020042 |
| KEGG_PATHWAY | cfa04080:Neuroactive ligand-receptor interaction | 0.030598685 |
| KEGG_PATHWAY | cfa05132:Salmonella infection | 0.030973107 |
| KEGG_PATHWAY | cfa05222:Small cell lung cancer | 0.038735701 |
| KEGG_PATHWAY | cfa05215:Prostate cancer | 0.043576819 |
| KEGG_PATHWAY | cfa04975:Fat digestion and absorption | 0.043703331 |
